# USP12 promotes CD4^+^ T cell responses through deubiquitinating and stabilizing BCL10

**DOI:** 10.1038/s41418-021-00787-y

**Published:** 2021-05-03

**Authors:** Yuling Fu, Peng Wang, Jingjing Zhao, Yunke Tan, Junli Sheng, Shitong He, Xialin Du, Yulan Huang, Yalong Yang, Jinling Li, Yuxiong Cai, Yuxuan Liu, Shengfeng Hu

**Affiliations:** 1 Institute of Molecular Immunology, School of Laboratory Medicine and Biotechnology, Southern Medical University, Guangzhou, China; 2 Department of Emergency Medicine, Sun Yat-Sen Memorial Hospital, Sun Yat-Sen University, Guangzhou, China; 3 Department of Biotherapy, Sun Yat-Sen University Cancer Center, Guangzhou, China; 4 State Key Laboratory of Oncology in South China, Collaborative Innovation Center for Cancer Medicine, Sun Yat-Sen University Cancer Center, Guangzhou, China

**Keywords:** Ubiquitins, T cells

## Abstract

Deubiquitinases (DUBs) regulate diverse biological processes and represent a novel class of drug targets. However, the biological function of only a small fraction of DUBs, especially in adaptive immune response regulation, is well-defined. In this study, we identified DUB ubiquitin-specific peptidase 12 (USP12) as a critical regulator of CD4^+^ T cell activation. USP12 plays an intrinsic role in promoting the CD4^+^ T cell phenotype, including differentiation, activation, and proliferation. Although USP12-deficient CD4^+^ T cells protected mice from autoimmune diseases, the immune response against bacterial infection was subdued. USP12 stabilized B cell lymphoma/leukemia 10 (BCL10) by deubiquitinating, and thereby activated the NF-κB signaling pathway. Interestingly, this USP12 regulatory mechanism was identified in CD4^+^ T cells, but not in CD8^+^ T cells. Our study results showed that USP12 activated CD4^+^ T cell signaling, and targeting USP12 might help develop therapeutic interventions for treating inflammatory diseases or pathogen infections.

## Introduction

T cells play a central role in adaptive immunity and are involved in fighting infection and tumorigenesis, as well as in the pathogenesis of autoimmune and inflammatory diseases [1–3]. The T cell receptor (TCR) signaling response, involved in regulating T cell activation and function, is essential for the generation of a T cell response [4]. After encountering the cognate peptide major histocompatibility complex on the surface of antigen-presenting cells (APCs) [5], TCR initiates TCR-proximal signaling, including tyrosine–protein kinase ζ-chain associated protein 70 (ZAP-70), linker for activation of T cells (LAT), and phospholipase C-γ1 (PLCγ1). This leads to the activation of a series of downstream signaling pathways, such as the NF-κB, EKR, and AKT signaling pathways, thus regulating the proliferation and differentiation of T cells during the immune response [6–9].

NF-κB is one of the key transcription factors involved in the TCR signaling pathway and is a central regulator of several genes involved in T cell survival, proliferation, and effector function [10, 11]. Thus, NF-κB activation is a tightly controlled process, in which the domain-containing membrane-associated guanylate kinase protein 1 (CARMA1)-B cell lymphoma/leukemia 10 (BCL10)-mucosa-associated lymphatic tissue 1 (MALT1) (CARMA1–BCL10–MALT1 (CBM)) complex plays a vital role [12]. Following TCR engagement, CARMA1 is activated by protein kinase Cθ and is recruited to the constitutive BCL10-MALT1 complex to form a CBM complex [13, 14]. The CBM complex then activated the downstream NF-κB signaling pathway by inducing proteasomal degradation of IκB proteins. NF-κB dissociates subsequently and is translocated to the nucleus to regulate NF-κB target gene expression [15, 16]. Interestingly, functional defects in the CBM complex impair NF-κB activation predominantly and subsequent downstream functional CD4^+^ T cell responses but not CD8^+^ T cells [17].

Immune cell signal transduction requires stringent regulation, which is subject to posttranslational modifications [18]. Ubiquitination is a crucial posttranslational modification that regulates diverse biological processes, including immune responses [19]. The primary role of ubiquitination is to target proteins to the proteasome for degradation, although several nondegradative functions have also been characterized [20, 21]. Ubiquitination is a reversible reaction. Ubiquitin chains are conjugated by the sequential action of three enzymes, E1 ubiquitin-activating enzyme, E2 ubiquitin-conjugating enzyme, and E3 ubiquitin ligase [22], and can also be cleaved by a large family of ubiquitin-specific proteases, termed deubiquitinases (DUBs) [23]. Deubiquitylation is involved in regulating both innate and adaptive immune responses [23]. DUBs exhibit specificity for both protein substrates and ubiquitin chains for differentially regulating protein ubiquitination. DUBs regulate diverse biological processes and represent a class of drug targets [24, 25]. However, the biological functions of only a limited number of DUBs are described to date, especially in regulating the adaptive immune response.

USP12, a DUB of the ubiquitin-specific protease (USP) family, has been reported to play a critical role in prostate cancer, HeLa, macrophage, and neuronal cells [26–29]. A previous in vitro study showed that USP12 stabilizes the TCR complex for the duration of signaling [30]. However, the in vivo activity of USP12 and its underlying mechanisms in T cell activation and differentiation remain unclear. This study aimed to investigate the molecular mechanisms underlying the USP12 regulation of T cell effector functions. Our results showed that USP12 activates CD4^+^ T cell but not CD8^+^ T cell proliferation through an intrinsic T cell function. USP12 deficiency caused BCL10 degradation leading to reduced NF-κB activation. Although USP12 activated CD4^+^ T cell responses against bacterial infections, it also promoted autoimmune disease development. Our results suggest that USP12 is an important target for regulating the adaptive immune response.

## Materials and methods

### Mice

C57BL/6 mice (wild type, WT) were from the Lab Animal Center of Southern Medicine University (Guangzhou, China). USP12-deficient (*Usp12*^−/−^), *Bcl10*^−/−^ mice, and *Uaf*1^*fl/fl*^ (*Wdr48*^*fl/fl*^) mice were built by Cyagen Biosciences Inc. (Guangzhou, China). CD4-Cre mice were from the Shanghai Research Center for Model Organisms (Shanghai, China). *Usp12*^−/−^ mice were crossed with and *Bcl10*^−/−^ mice to generate *Usp12*^−/−^ *Bcl10*^−/−^ mice. OT-I and OT-II mice were from The Jackson Laboratories. CD45.1^+^ and *Rag1*^−/−^ mice were purchased from Nanjing Biomedical Research Institute (Nanjing, China). OT-II or OT-I mice were crossed with *Usp12*^−/−^ mice to generate *Usp12*^−/−^ OT-II and WT OT-II control mice or *Usp12*^−/−^ OT-I and WT OT-I control mice. *Uaf*1^*fl/fl*^ mice were crossed with CD4-Cre mice to generate *Uaf*1^*fl/fl*^ mice, CD4-Cre (*Uaf1*^*CKO*^) mice. All mice were all C57BL/6 background and maintained in the Lab Animal Center of Southern Medicine University under specific pathogen-free conditions. All animal experiments were conducted in accordance with protocols approved by the Medical Ethics Board and the Biosafety Management Committee of Southern Medical University (approval number SMU-L2016022). All mice were used at an age of 6–12 weeks and were randomly divided into different groups.

### EAE model

WT, *Usp12*^−/−^, *Bcl10*^−/−^, *Usp12*^−/−^ *Bcl10*^−/−^, and recipient *Rag1*^−/−^ mice reconstituted by WT or *Usp12*^−/−^ CD4^+^ T cells were immunized subcutaneously with 200 μg MOG (35–55) peptide emulsified in CFA (Difco Laboratories, USA) with 400 μg *Mycobacterium tuberculosis* H37Ra on day 0. To induce experimental autoimmune encephalomyelitis (EAE) development and assess the severity of EAE, mice also received 200 ng of pertussis toxin (Sigma, USA) by intraperitoneal injection on days 0 and 2. Symptoms of EAE were monitored daily using a classical clinical score ranging from 0 to 5 as follows: 0, no disease; 1, tail paralysis; 2, weakness of hind limbs; 3, paralysis of hind limbs; 4, paralysis of hind limbs and severe hunched posture; 5, moribund or death, as previously described [31].

### *L. monocytogenes* infection

WT, *Usp12*^−/−^ mice, and recipient *Rag1*^−/−^ mice were infected i.v. with a recombinant *Listeria monocytogenes* (*L. monocytogenes*) strain expressing chicken ovalbumin, LM-OVA (5 × 10^4^ colony-forming unit (CFU) per mouse) and sacrificed after 7 days of infection to analyze the primary host response. Livers were homogenized in 10 ml 0.2% (vol/vol) Nonidet P-40 in PBS, and the organ homogenates were serially diluted and plated on streptomycin agar plates to determine the CFU of *L. monocytogenes*. Splenocytes were collected and restimulated by 10 μg/ml of OVA (323–339) for flow cytometry analysis.

### Flow cytometry analysis

For intracellular cytokine staining (ICS) assays, T cells isolated from spleen of mice or from in vitro cultures were stimulated for 1.5 h with 100 mg/ml MOG(35-55) or PMA (50 ng/ml, Thermo Fisher Scientific, USA) and ionomycin (500 ng/ml, Thermo Fisher Scientific), before Brefeldin A (10 μg/ml, eBioscience, USA) was added to the culture for 3.5 h more. As previously described [32], for surface staining, cells were harvested, washed, and stained for 30 min on ice with mixtures of fluorescently conjugated mAbs or isotype-matched controls. For ICS, cells were stained for surface molecules, fixed 20 min in IC Fixation buffer (Thermo Fisher Scientific), and incubated for 1 h in permeabilization buffer (Thermo Fisher Scientific) with appropriate mAbs of mice. Antibodies used in this study are listed in Supplementary Table 1. Cell phenotype was analyzed by flow cytometry on a flow cytometer (BD LSR II) (BD Biosciences, USA) or Attune NxT (Thermo Fisher Scientific). Data were acquired as the fraction of labeled cells within a live-cell gate and analyzed using FlowJo software (Tree Star). All gates were set on the basis of isotype-matched control antibodies.

### MOG(35-55) recall assay

Splenocytes were isolated from mice induced EAE, were restimulated with 100 μg/ml MOG (35–55) incomplete RPMI1640 media for 48 h. Cell culture supernatants were collected for enzyme-linked immunosorbent assay (ELISA).

### Naïve T cell isolation and T cell activation assay in vitro

Spleen and lymph node cells were isolated from mice. CD4^+^ T or CD8^+^ T cells were negatively selected using EasySep^TM^. Mouse Naive CD4^+^ T or CD8^+^ T cell Isolation Kit (Miltenyi, Germany). Purified naive T cells were stimulated with plate-bound anti-CD3 (1 μg/ml or indicated concentrations) and soluble anti-CD28 antibodies (1 μg/ml) in replicate wells of 96-well plates (1 × 10^5^ cells per well) for flow cytometry analysis and ELISA, 12-well plates (1 × 10^6^ cells per well) for quantitative polymerase chain reaction (qPCR) and 6-well plates (5 × 10^6^ per well) for western blot assays.

### Enzyme-linked immunosorbent assay

Cytokine production in supernatants of in vitro cell cultures or sera of mice was measured by ELISA of mouse IFN-γ, IL-17, TNF-α, and IL-2 (ExCell Bio, China) according to the manufacturer’s protocol.

### Thymidine incorporation assay

Purified naive T cells were stimulated with increasing concentrations of plate-bound anti-CD3 and anti-CD28in triplicate wells for 48 h. During the last 8 h of stimulation, T cells were pulsed with [^3^H] thymidine and the amount of incorporated [^3^H] thymidine was measured as counts per minute (CPM). For signal pathway-inhibition studies, the NF-κB-inhibitor SC514 (3 mg/ml) (Selleck, USA) was added into culture media.

### CFSE T cell proliferation assay

Purified naïve T cells were labeled with 2.5 μM CFSE and then 5 × 10^4^ T cells/well were stimulated with anti-CD3 and anti-CD28. T cells were cultured for 72 h and proliferation was determined by flow cytometry analysis of carboxyfluorescein succinimidyl ester (CFSE) dilution.

### CD4^+^ T cell differentiation

Purified naive CD4^+^ T cells were stimulated with plate-bound anti-CD3 and anti-CD28 under Th1 (10 ng/ml IL-12 and 10 μg/ml anti-IL4, Peprotech, USA), Th2 (20 ng/ml IL-4 and 10 μg/ml anti-IFN-γ, Peprotech), Th17 (2.5 ng/ml TGF-β, 15 ng/ml IL-6, 10 μg/ml anti-IFN-γ, and 10 μg/ml anti-IL4, Peprotech), and Treg (1.5 ng/ml TGF-β, 10 μg/ml anti-IFN-γ, and 10 μg/ml anti-IL4, Peprotech) conditions. After 5 days of stimulation, the cells were subjected to ELISA or qPCR analyses.

### Quantitative PCR (qPCR) analysis

Total RNA was isolated with Trizol (Thermo Fisher Scientific) according to the manufacturer’s instructions. A 1 mg of RNA was reverse transcribed to cDNA with random RNA-specific primers using the high-capacity cDNA reverse transcription kit (Applied Biosystems, USA). An Eppendorf Master Cycle Realplex2 and an SYBR Green PCR Master Mix (Applied Biosystems) were used for real-time PCR (40 cycles). The primer sequences used for PCR are in Supplementary Table 2.

### DC purification and DC-T cell coculture

Bone marrow cells isolated from the tibias and femurs of naïve mice were cultured for 7 days in the presence of the cytokine granulocyte-macrophage colony-stimulating factor (20 ng/ml; Peprotech) and IL-4 (10 ng/ml; Peprotech). On day 7, cells were purified with CD11c^+^ magnetic beads (Miltenyi). DCs were activated for 48 h with lipopolysaccharides. DCs were pulsed with OVA (323–339) peptides (100 ng/ml, Sigma) for 2 h and used (at a ratio of 1:10) to stimulate naive CD4^+^ T cells from WT or *Usp12*^−/−^ OT-II mice for the indicated time. In the same way, DCs were pulsed with OVA (257–264) peptides and used to stimulate naive CD8^+^ T cells from WT or *Usp12*^−/−^ OT-I mice.

### Immunoblot, co-immunoprecipitation, and ubiquitination assays

The experiments were performed as previously described [33, 34]. Spinal cords or cells were washed three times with ice-cold PBS and then lysed in Nonidet P-40 lysis buffer containing 150 mM NaCl, 1 mM EDTA, 1% Nonidet P-40, and 1% protease and phosphatase inhibitor cocktail (Biotool). Equal amounts (20 mg) of cell lysates were resolved using 8 ± 15% polyacrylamide gels transferred to polyvinylidene fluoride membrane. Membranes were blocked in 5% non-fat dry milk in PBST and incubated overnight with the respective primary antibodies at 4 °C. The membranes were incubated at room temperature for 1 h with appropriate horseradish peroxidase-conjugated secondary antibodies and visualized with Plus-ECL (PerkinElmer, CA) according to the manufacturer’s protocol. For immunoprecipitation assays, the lysates were immunoprecipitated with IgG or the appropriate antibodies and protein G Sepharose beads. The precipitates were washed three times with lysis buffer containing 500 mM NaCl, followed by immunoblot analysis. For deubiquitination assays, the cells were lysed with the lysis buffer and the supernatants were denatured at 95 °C for 5 min in the presence of 1% SDS. The denatured lysates were diluted with lysis buffer to reduce the concentration of SDS below 0.1% followed by immunoprecipitation with the indicated antibodies. The immunoprecipitates were subjected to immunoblot analysis with anti-ubiquitin chains. Antibodies used in this study are listed in Supplementary Table 1.

### Metabolism assays

OCR and ECAR were measured with an XF96 extracellular flux analyzer (Seahorse Bioscience, USA) following the manufacturer’s instructions. In brief, CD4^+^ or CD8^+^ T cells were isolated from WT and *Usp12*^−/−^ mice, stimulated with anti-CD3 and anti-CD28 antibodies with or without the NF-kB inhibitor (SC154) for 24 h, and seeded in XF96 microplates. The plates were quickly centrifuged to immobilize the cells. After incubation in a non-buffered assay medium (Seahorse Biosciences) in an incubator without CO_2_ for 30 min, the cells were subjected to measure ECAR or OCR with an XF glycolysis stress test kit or Mito stress test kit. ECAR and OCR rates were assessed at basal conditions and after the addition of glucose (10 mM), oligomycin (1 mM), and 2-Deoxyglucose (2-DG, 20 mM) for ECAR, and after the addition of oligomycin (1 mM), FCCP (1 mM), and rotenone (1 mM) for OCR. Compounds were added at the indicated time points and the assay was performed using a Seahorse Extracellular XF96 analyzer. Three measurements were recorded for basal metabolic rates and following each injection.

### Retroviral packaging and transduction

Genes encoding wild-type USP12, USP12 (C48A), USP46, or USP9X were cloned into retroviral vector pMXs containing IRES-regulated GFP (Youbio, China), respectively. Each of the resulting plasmids was transfected into a packaging cell line, PLAT-T, using FuGENE6 (Roche, Switzerland). After incubation for 24 h, the culture supernatant was harvested and condensed as a viral stock. The CD4^+^ T cells were stimulated by anti-CD3 and anti-CD28 antibodies for 24 h. The cells were then infected with retrovirus in the presence of 0.5 μg/ml of polybrene for 24 h and cultured further in the presence of 30 U/ml of IL-2 for 3 days. The cells were washed with fresh media and restimulated by anti-CD3/anti-CD28 antibodies for 6 h for ICS and for 24 h for the cytokine production assay.

### Statistics

All experiments were performed at least thrice. When shown, multiple samples represent biological (not technical) replicates of mice randomly sorted into each experimental group. No blinding was performed during animal experiments. Determination of statistical differences was performed with Prism 8 (Graphpad Software, Inc.) using unpaired two-tailed *t* tests (to compare two groups with similar variances), or two-way ANOVA with Bonferonni’s multiple comparison test (to compare more than two groups).

## Results

### USP12-deficiency attenuated in vivo CD4^+^ T cell activation

The ability of USP12 to modulate T cell responses and the underlying mechanisms are unclear. The role of USP12 in T cell function was investigated using USP12-knockout (*Usp12*^−/−^) mice. USP12 immunoblot results using splenocytes from wild-type (WT) and *Usp12*^−/−^ mice showed the lack of USP12 protein in *Usp12*^−/−^ mice (Supplementary Fig. 1A). The *Usp12*^−/−^ mice showed expected Mendelian ratios and a comparable survival rate as WT control mice (Supplementary Fig. 1B). *Usp12*^−/−^ mice did not show any distinct abnormalities in thymocyte development or peripheral T and B cell homeostasis (Supplementary Fig. 1C–G).

**Fig. 1:**
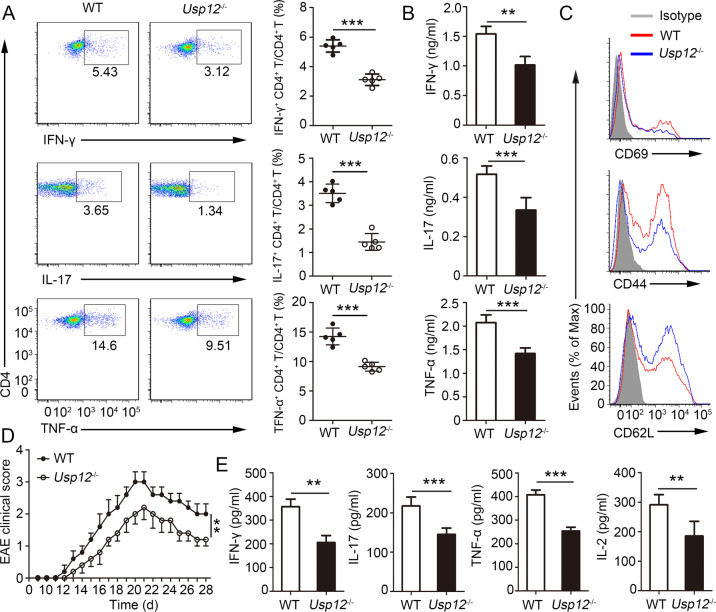
USP12 deficiency attenuated the generation of hyperinflammatory CD4^+^ T cell responses in vivo. **A**–**C** WT and *Usp12*^−/−^ mice were immunized with MOG (35–55) peptide in CFA adjuvant and mice were harvested on day 20. **A** Splenocytes were restimulated directly ex vivo and the intracellular production of IFN-γ, IL-17, and TNF-α by CD4^+^ T cells was determined. Pooled data are presented in the right panel. **B** Splenocytes were stimulated for 48 h with MOG(35–55) peptide and cytokine production was measured by ELISA. **C** Expression of activation markers by splenic CD4^+^ T cells. **D**, **E** WT and *Usp12*^−/−^ mice were immunized with MOG (35–55) peptide in CFA adjuvant and pertussis toxin to induce EAE. **D** The graph shows the clinical score of EAE (*n* = 10 for WT mice and *Usp12*^−/−^ mice, respectively). **E** Mice were harvested on day 28 and concentration of IFN-γ, IL-17, TNF-α, and IL-2 in serum was measured by ELISA. Data (*n* = 5 in A, B, and E) shown are the mean ± SD. **P < 0.01 and ***P < 0.001 by an unpaired *t*-test. Data are representative of three independent experiments with similar results.

The role of USP12 in shaping T cell responses was elucidated by immunizing WT and *Usp12*^−/−^ mice with MOG (35–55) peptide in CFA adjuvant. CD4^+^ T cells from *Usp12*^−/−^ mouse spleen showed decreased intracellular IFN-γ, TNF-α, and IL-17A expression after MOG (35–55) peptide stimulation compared to CD4^+^ T cells from WT mouse spleen (Fig. 1A). After stimulation with MOG (35–55) peptide, *Usp12*^−/−^ splenocytes secreted higher levels of cytokines (Fig. 1B). Activation analysis showed that CD4^+^ T cells from *Usp12*^−/−^ mice expressed reduced CD69 and CD44 levels and markedly upregulated CD62L levels relative to WT CD4^+^ T cells (Fig. 1C and Supplementary Fig. 2A). However, USP12 deficiency did not affect the proportion of Th2 and Treg cells (CD25^+^ Foxp3^+^) in CD4^+^ T cells (Supplementary Fig. 2B, C), and the intracellular IFN-γ and TNF-α expression in CD8^+^ T cells (Supplementary Fig. 2D). Consistently, CD4^+^ T cells in USP12-deficient conditions exhibited a reduced activation ability; thus, USP12-deficiency restricts EAE progression (Fig. 1D). The *Usp12*^−/−^ mouse sera contained lower IFN-γ and TNF-α levels than the WT mouse sera (Fig. 1E).

Although excess CD4^+^ T cell response leads to autoimmune diseases, reduced CD4^+^ T cell activation does not protect the body from pathogen infections. Hence, we used a recombinant *L. monocytogenes* strain expressing chicken ovalbumin (LM-OVA) infection model to investigate the USP12 knockdown effects on the CD4^+^ T cell phenotype. The *Usp12*^−/−^ mice displayed markedly reduced immune responses against LM-OVA infection, as demonstrated by fewer IFN-γ producing CD4^+^ T cells (Supplementary Fig. 2E), reduced survival rate (Supplementary Fig. 2F), increased bacterial load in the liver (Supplementary Fig. 2G), and reduced IFN-γ and TNF-α serum levels (Supplementary Fig. 2H) compared to the WT mice. Altogether, the above results show that USP12 is a critical regulator of CD4^+^ T cell activation.

### USP 12 is required for in vitro CD4^+^ T cell activation

**Fig. 2:**
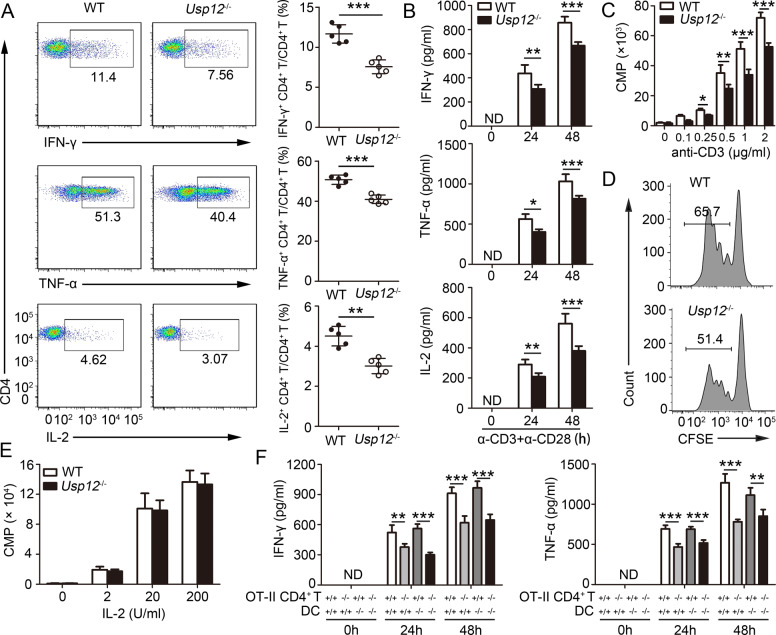
CD4^+^ T cell activation in vitro required USP12. **A**–**E** Purified naïve CD4^+^ T cells isolated from WT and *Usp12*^−/−^ mice were either not treated **(0)** or stimulated with plate-bound anti-CD3 (1 μg/ml or indicated concentrations) and anti-CD28 (1 μg/ml) for 48 h (**A**, **C**, **E**) or 72 h (**D**) or indicated amounts of time (**B**). **A** The intracellular production of IFN-γ, TNF-α, and IL-2 by CD4^+^ T cells was determined. Pooled data are presented in the right panel. **B** Cytokine production was measured by ELISA. **C** The incorporation of thymidine was measured during the final 8 h. **D** Isolated purified naïve CD4^+^ T cells were labeled with CFSE, stimulated and determined by flow cytometry. **E** Purified WT and *Usp12*^−/−^ naive CD4^+^ T cells first stimulated with anti-CD3 and CD28 and then cultured with various concentrations of IL-2. The incorporation of thymidine was measured for 8 h. **F** OVA (323–339) coated DCs from WT and *Usp12*^−/−^ mice were cocultured with OT-II CD4^+^ T cells from WT OT-II or *Usp123* OT-II mice for indicated amounts of time. IFN-γ and TNF-α production was measured by ELISA+/+: WT; −/−: *Usp12*^−/−^. Data (*n* = 5 in **A**–**F**) shown are the mean ± SD. **P < 0.01 and ***P < 0.001 by an unpaired *t* test (in **A**) or two-way ANOVA (in **B**, **C**, **E,** and **F**). Data are representative of three independent experiments with similar results. ND no detected.

We determined whether USP12 modulates CD4^+^ T cell activation through an intrinsic role in T cell function or an extrinsic role in APC. First, we hypothesized that USP12 is a direct regulator of CD4^+^ T cells. To confirm this hypothesis, naïve CD4^+^ T cells from WT and *Usp12*^−/−^ mice were activated in vitro. We found that USP12 deficiency reduced IFN-γ, TNF-α, and IL-2 production in CD4^+^ T cells, as assessed by ICS (Fig. 2A) and ELISA (Fig. 2B). Moreover, *Usp12*^−/−^ CD4^+^ T cells showed reduced proliferation based on thymidine incorporation (Fig. 2C) or CFSE dye dilution methods (Fig. 2D and Supplementary Fig. 3A, B). IL-2 is a key cytokine that affects CD4^+^ T cell proliferation; hence, we speculated whether decreased IL-2 production is responsible for reduced *Usp12*^−/−^ CD4^+^ T cell proliferation. We investigated this by stimulating CD4^+^ T cells with anti-CD3 and anti-CD28 antibodies, and, after the cells were washed, they were cultured with exogenous IL-2. There was no marked difference in the proliferation of WT and *Usp12*^−/−^ CD4^+^ T cells (Fig. 2E). We examined whether USP12 modulates CD4^+^ T cell activation through an extrinsic role in APC, WT OT-II, and *Usp12*^−/−^ OT-II by co-culturing CD4^+^ T cells with either WT or *Usp12*^−/−^ dendritic cells (DCs) pulsed with OVA (323–339). The IFN-γ, TNF-α, and IL-2 levels were significantly lower in cultures of *Usp12*^−/−^ OT-II CD4^+^ T cells compared to those of WT OT-II CD4^+^ T cells, irrespective of the genotype of the DCs (Fig. 2F and Supplementary Fig. 3C). Next, we examined USP12 role in regulating CD4^+^ T cell differentiation. Under standard CD4^+^ T cell differentiation conditions, WT and *Usp12*^−/−^ CD4^+^ T cells showed similar transcription factor expression (Supplementary Fig. 3D), although IFN-γ and IL-17 mRNA expression was downregulated in *Usp12*^−/−^ CD4^+^ T cells (Supplementary Fig. 3E). Previous in vivo results showed that USP12-deficiency did not affect CD8^+^ T cell activation. We evaluated whether USP12 modulates in vitro CD8^+^ T cell activation and, consistent with the in vivo results, USP12 deficiency did not affect CD8^+^ T cell activation, as assessed by ICS (Supplementary Fig. 4A) and ELISA (Supplementary Fig. 4B), thymidine incorporation (Supplementary Fig. 4C), and CFSE T cell proliferation assays (Supplementary Fig. 4D, E). Results from coculturing WT OT-I and *Usp12*^−/−^ OT-I CD8^+^ T cells with either WT or *Usp12*^−/−^ DCs that were pulsed with OVA (257–264), showed that USP12 deficiency in DCs did not affect CD8^+^ T cell activation (Supplementary Fig. 4F). Collectively, these results suggest that USP12 stimulates naïve CD4^+^ T cell activation through an intrinsic T cell function.

### USP12 is an intrinsic regulator of in vivo CD4^+^ T cell activation

**Fig. 3:**
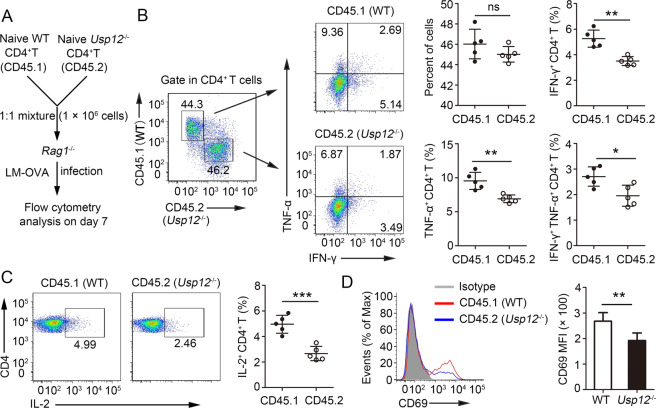
USP12 promoted CD4^+^ T cell activation in a T-cell-intrinsic manner in vivo. **A** Schematic of experimental design of competitive adoptive CD4^+^ T cell transfer assays. **B** Splenocytes were restimulated with OVA (323–339) directly ex vivo and the intracellular production of IFN-γ and TNF-α by CD4^+^ T cells was determined. Representative FACs plots depicting gating of CD4^+^ T cells are shown. Gating strategies to evaluate cytokine production by WT and *Usp12*^−/−^ CD4^+^ T cells are provided. Numbers in the quadrants indicate the percent cells in each. Pooled data are presented in the right panel. **C** Splenocytes were restimulated with OVA (323–339) directly ex vivo and the intracellular production of IL-2 by CD4^+^ T cells was determined. Pooled data are presented in the right panel. **D** Expression of CD69 by splenic CD4^+^ T cells was determined by flow cytometry. Pooled data of mean fluorescence intensity (MFI) are presented in the right panel. Data (*n* = 5 in **B**–**D**) shown are the mean ± SD. *P < 0.05 and **P < 0.01 by an unpaired *t* test. Data are representative of three independent experiments with similar results.

To further confirm the intrinsic role of USP12 in regulating CD4^+^ T cell responses, we conducted in vivo competitive adoptive CD4^+^ T cell transfer assays. For these experiments, *Rag1*^−/−^ recipient mice received 1:1 CD45.1^+^ WT and CD45.2^+^ *Usp12*^−/−^ naïve CD4^+^ T cells and were infected subsequently with LM-OVA (Fig. 3A). Although there was no significant difference in CD4^+^ T cell percentage between CD45.1^+^ WT and CD45.2^+^ *Usp12*^−/−^ CD4^+^ T cells in the same environment (Fig. 3B), CD45.2^+^ *Usp12*^−/−^ CD4^+^ T cells had considerably higher IFN-γ and TNF-α producing cells than CD45.1^+^ WT CD4^+^ T cells (Fig. 3B). Likewise, the percentage of CD4^+^ T cells producing IL-2 was higher in CD45.2^+^ *Usp12*^−/−^ CD4^+^ T cells than in CD45.1^+^ WT CD4^+^ T cells (Fig. 3C). Activation analysis showed upregulated CD69 expression in CD45.2^+^ *Usp12*^−/−^ CD4^+^ T cells compared to that in CD45.1^+^ WT CD4^+^ T cells (Fig. 3D). Together, these results show USP12 as an intrinsic regulator of CD4^+^ T cell activation.

### USP12 deficiency in CD4^+^ T cells attenuates in vivo immune response

**Fig. 4:**
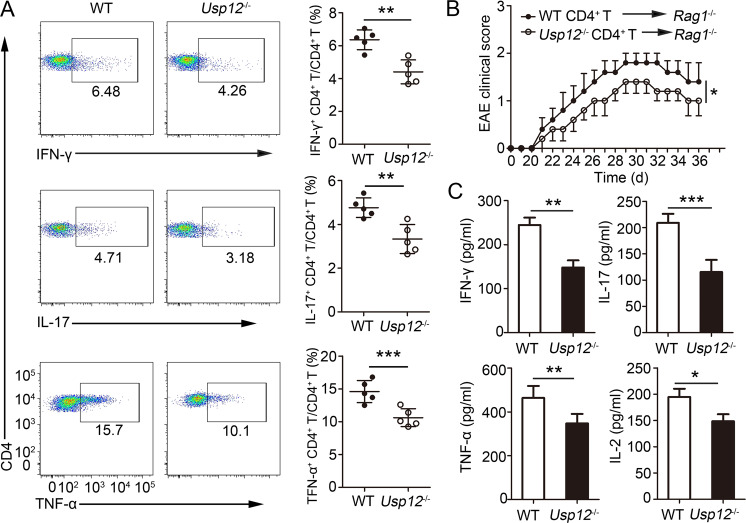
USP12 deficiency in CD4^+^ T cells attenuated the immune response in vivo. Purified WT or *Usp12*^−/−^ naïve CD4^+^ T cells were adoptively transferred into *Rag1*^−/−^ mice. **A** One day later, the recipient mice were immunized with MOG (35–55) peptide in CFA adjuvant. Splenocytes were restimulated directly ex vivo and the intracellular production of IFN-γ, IL-17, and TNF-α by CD4^+^ T cells was determined. Pooled data are presented in the right panel. **B**, **C** One day later, the recipient mice were immunized with MOG (35–55) peptide in CFA adjuvant and pertussis toxin to induce EAE. **B** The graph shows the clinical score of EAE. **C** Mice were harvested on day 36 and concentration of IFN-γ, IL-17, TNF-α, and IL-2 in serum was measured by ELISA. Data (*n* = 5 in **A**–**C**) shown are the mean ± SD. **P < 0.01, **P < 0.01, and ***P < 0.001 by an unpaired *t* test. Data are representative of three independent experiments with similar results.

To understand the impact of USP12-deficient CD4^+^ T cells on the in vivo immune response, *Rag1*^−/−^ recipient mice were administered WT and *Usp12*^−/−^ naïve CD4^+^ T cells and were subsequently immunized with MOG (35–55) peptide in CFA adjuvant. In experiments to detect the activation and development of CD4^+^ T cells, we observed that CD4^+^ T cells isolated from the spleen of *Rag1*^−/−^ mice that received *Usp12*^−/−^ CD4^+^ T cells expressed reduced intracellular IFN-γ, IL-2, and TNF-α after stimulation with MOG (35–55) peptide compared to mice that received WT CD4^+^ T cells (Fig. 4A). *Rag1*^−/−^ mice receiving *Usp12*^−/−^ CD4^+^ T cells developed significantly attenuated EAE compared to those receiving WT CD4^+^ T cells (Fig. 4B). The cytokine levels were also lower in the serum of *Rag1*^−/−^ recipients receiving *Usp12*^−/−^ CD4^+^ T cells than in the serum of those receiving WT CD4^+^ T cells (Fig. 4C). Furthermore, we determined USP12-deficient CD4^+^ T cells’ role in eliciting an immune response using a bacterial model. We found that *Rag1*^−/−^ mice receiving *Usp12*^−/−^ CD4^+^ T cells showed decreased intracellular IFN-γ expression in the CD4^+^ T cells of the spleen (Supplementary Fig. 5A), reduced survival rate (Supplementary Fig. 5B), increased bacterial load in the liver (Supplementary Fig. 5C), and reduced IFN-γ and TNF-α levels in the serum (Supplementary Fig. 5D) compared to *Rag1*^−/−^ mice receiving WT CD4^+^ T cells. These results suggest an intrinsic USP12 function for regulating the immune response in CD4^+^ T cells.

### USP12 regulates NF-κB signaling in activated CD4^+^ T cells

**Fig. 5:**
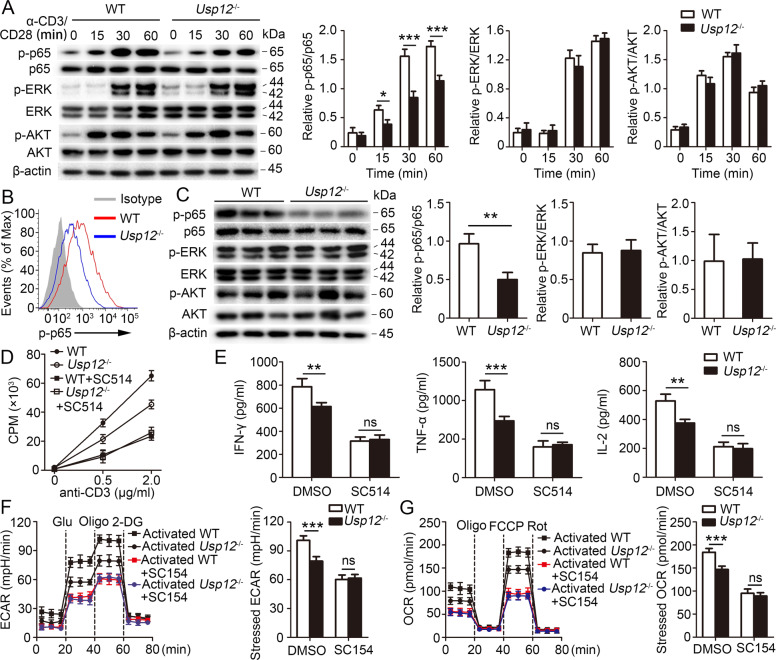
USP12 promoted the NF-κB signaling pathway in CD4^+^ T cells. **A** Western blot analyses of purified naïve WT and *Usp12*^−/−^ CD4^+^ T cells stimulated by anti-CD3 and anti-CD28 antibodies. Densitometry quantification of band intensity are presented in the right panel. **B** Flow cytometry analyses of phosphorylated p65 in CD4^+^ T cells sorted from spleen of WT and *Usp12*^−/−^ mice immunized with MOG (35–55) peptide in CFA adjuvant and harvested on day 20. **C** Western blot analyses of spinal cords from CD4^+^ T-cell-transferred *Rag1*^−/−^ mice 20 days after induction of EAE. Each lane represents a spinal cord from a different mouse. Densitometry quantification of band intensity are presented in the right panel. **D**–**G** Purified naïve CD4^+^ T cells from WT or *Usp12*^−/−^ mice were stimulated with anti-CD3 (1 μg/ml or indicated concentrations) and anti-CD28 antibodies together with NF-kB inhibitor SC514 (3 mg/ml) or DMSO. **D** Counts per minute (CPM) of CD4^+^ T cells at 48 h after stimulation with anti-CD3 and anti-CD28. **E** Secretion of IFN-γ, TNF-α, and IL-2 at 48 h after stimulation. **F** Seahorse analysis of ECAR at 24 h after stimulation. Data are presented as a representative plot and summary graphs. **G** Seahorse analysis of OCR at 24 h after stimulation. Data are presented as a representative plot and summary graphs. Data (*n* = 3 in **A**–**C**, *n* = 5 in **D–G**) shown are the mean ± SD. *P < 0.05, **P < 0.01, and ***P < 0.001 by an unpaired *t* test (in **C**) and two-way ANOVA (in **A**, **D**–**G**). Data are representative of three independent experiments with similar results.

Next, we explored USP12-controlled specific molecular pathways in CD4^+^ T cells. Upon stimulation with anti-CD3 and anti-CD28 antibodies, WT and *Usp12*^−/−^ CD4^+^ T cells displayed similar TCR-proximal signaling events, including phosphorylation of ZAP70, LAT, and PLCγ1 (Supplementary Fig. 6A), and downstream signaling events, including phosphorylation of AKT and ERK (Fig. 5A). However, *Usp12*^−/−^ CD4^+^ T cells showed reduced p65 phosphorylation, another TCR downstream signaling event (Fig. 5A). Then we analyzed whether USP12 regulates the NF-κB signal pathway in CD4^+^ T cells in vivo, and found a significant difference in p65 phosphorylation between WT and *Usp12*^−/−^ CD4^+^ T cells and no difference in AKT and ERK phosphorylation (Fig. 5B and Supplementary Fig. 6B). To further assess whether USP12 regulates NF-κB signaling in CD4^+^ T cells, we transferred CD4^+^ T cells from WT or *Usp12*^−/−^ mice into *Rag1*^−/−^ mice, which were later administered MOG (35–55) peptide. Our results showed that the transfer of *Usp12*^−/−^ CD4^+^ T cells resulted in enhanced p65 phosphorylation in the spinal cords of recipient mice (Fig. 5C), although AKT and ERK phosphorylation was not different between the two groups (Fig. 5C). After treatment with SC514, a pharmacological inhibitor of NF-κB activation, proliferation (Fig. 5D) and IFN-γ, TNF-α, and IL-2 expression (Fig. 5E) were not different between WT and *Usp12*^−/−^ CD4^+^ T cells.

Metabolic reprogramming is a hallmark of T cell activation and is required for effector T cell function. To investigate whether USP12 affects T cell metabolism, Seahorse extracellular flux analyses were conducted to measure the extracellular acidification rate (ECAR) and oxygen consumption rate (OCR). Compared to WT CD4^+^ T cells, *Usp12*^−/−^ CD4^+^ T cells had reduced ECAE and maximum glycolytic capacity (stressed ECAR) under activated conditions (Supplementary Fig. 6C). Similar to glycolysis, OCR, and maximum mitochondrial oxidative phosphorylation (stressed OCR) were also altered significantly in USP12-deficient CD4^+^ T cells (Supplementary Fig. 6D). Pharmacological inhibition assays showed that treatment of CD4^+^ T cells with the NF-κB inhibitor SC154 resulted in similar ECARs and OCRs between activated WT and *Us12*^−/−^ CD4^+^ T cells (Fig. 5E, F).

Furthermore, we investigated whether USP12 regulated the NF-κB signaling pathway in CD8^+^ T cells and found that USP12 deficiency in CD8^+^ T cells did not affect in vitro (Supplementary Fig. 7A) or in vivo (Supplementary Fig. 7B) NF-κB activation. Similarly, metabolic reprogramming in activated CD8^+^ T cells was unaltered by USP12, as demonstrated by similar ECAEs (Supplementary Fig. 7C) and OCRs (Supplementary Fig. 7D) in WT and *Usp12*^−/−^ CD8^+^ T cells. Altogether, these results indicated that USP12 activated CD4^+^ T cell response by targeting the NF-κB signaling pathway.

### USP12 regulates BCL10 stability

**Fig. 6:**
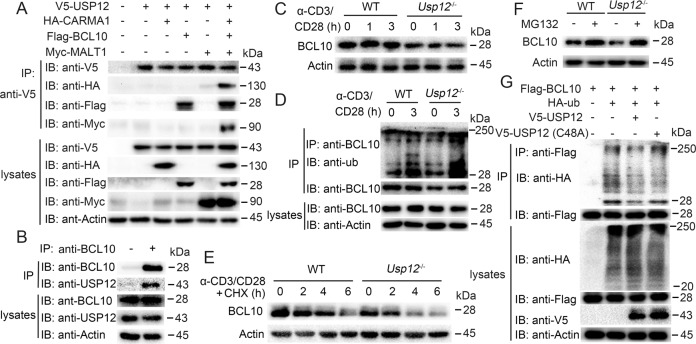
USP12 deubiquitinated and stabilized BCL10. **A** Immunoprecipitation (IP) and immunoblot (IB) analysis of HEK293 cells that were transfected with indicated plasmids for 24 h. **B** IP and IB of CD4^+^ T cells that were stimulated with anti-CD3 and anti-CD28 antibodies for 1 h. −: control lgG; +: anti-BCL10. **C**–**F** BCL10 IB (**C**, **E**, **F**) and ubiquitination (**D**) analysis using whole-cell extracts of WT or *Usp12*^−/−^ naive CD4^+^ T cells stimulated with anti-CD3and anti-CD28 for 3 h (**F**) or indicated time (**C–E**). MG132 was added (in **D** or +) or not added (−). **G** BCL10 ubiquitination assays using HEK293 cells transfected with expression vectors encoding indicated proteins for 24 h.

The CBM signalosome complex forms an essential molecular link between the cell-surface antigen receptors and triggers selective NF-κB activation in CD4^+^ T cells. Interestingly, USP12 deficiency also had a selective effect on CD4^+^ T cells; hence, we investigated whether USP12 acts by regulating the CBM complex in CD4^+^ T cells. By co-transfecting USP12 and the individual CBM complex protein into HEK293 cells, we showed USP12 interaction with BCL10 but not CARMA1 and MALT1 (Fig. 6A). In addition, endogenous USP12 and BCL10 also formed a stable complex within activated CD4^+^ T cells (Fig. 6B). USP12 deficiency resulted in a significant loss of BCL10 protein in CD4^+^ T cells, whether CD4^+^ T cells were stimulated or not (Fig. 6C). However, the protein levels of CARMA1 and MALT1 did not change with the USP12 deficiency (Supplementary Fig. 8A). Since USP12 is a DUB, we investigated whether USP12 acts through DUB activity. We found that USP12 deficiency potentiated BCL10 ubiquitination (Fig. 6D and Supplementary Fig. 8B) and promoted BCL10 degradation (Fig. 6E). BCL10 degradation in *Usp12*^−/−^ CD4^+^ T cells was completely blocked by the proteasome inhibitor MG132 (Fig. 6F). Consistent with the in vivo results, USP12, but not the enzymatically inactive mutant USP12(C48A), catalyzed BCL10 deubiquitination in HEK293 cells (Fig. 6G and Supplementary Fig. 8C). Overexpression of USP12 significantly inhibited the degradation of BCL10 in *Usp12*^−/−^ CD4^+^ T cells, but overexpression of USP12(C48A) did not (Supplementary Fig. 8D).

USP12 has been shown to have very limited deubiquitylating activity in the absence of other adapter proteins such as UAF1 (or WDR48) and WDR20 [35]. We checked whether USP12 requires the presence of these co-factors to mediate stabilization of BCL10 in CD4^+^ T cells, and found that USP12 interaction with UAF1 and WDR20 (Supplementary Fig. 9A, B). UAF1 deficiency in CD4^+^ T cells led to decreased BCL10 protein and IFN-γ expression, which was not completely retuned by overexpression of USP12 (Supplementary Fig. 9C, D). USP46 and USP12 are >80% homologous and have been proposed to be redundant in their functions [30]. Overexpression of USP46 in *Usp12*^−/−^ CD4^+^ T cells reversed the decrease in IFN-γ expression caused by USP12 deficiency (Supplementary Fig. 9E, F). USP9X has been previously shown to act on BCL10 to stabilize the CBM complex [12]. However, we found that USP9X expression was not affected by UPS12 deficiency (Supplementary Fig. 9G), and overexpression of USP9X in *Usp12*^−/−^ CD4^+^ T cells did not reverse the decrease in IFN-γ expression caused by USP12 deficiency (Supplementary Fig. 9H, I). Together, these data suggest that USP12 stabilizes BCL10 by deubiquitination in CD4^+^ T cells.

### USP12 regulates CD4^+^ T activation and function by targeting BCL10

**Fig. 7:**
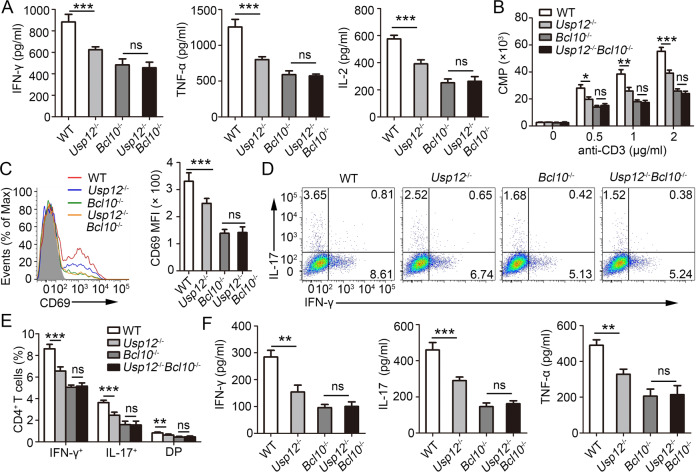
USP12 promoted CD4^+^ T cell activation-dependent on BCL10. **A**–**C** Purified naïve CD4^+^ T cells isolated from WT, *Usp12*^−/−^, *Bcl10*^−/−^, and *Usp12*^−/−^ *Bcl10*^−/−^ mice were stimulated with plate-bound anti-CD3 (1 μg/ml or indicated concentrations) and anti-CD28 for 48 h. **A** Cytokine production was measured by ELISA. **B** The incorporation of thymidine was measured during the final 8 h. **C** Expression of CD69 was determined by flow cytometry. Pooled data of mean fluorescence intensity (MFI) are presented in the right panel. **D**–**F** WT, *Usp12*^−/−^, *Bcl10*^−/−^, and *Usp12*^−/−^ *Bcl10*^−/−^ mice were immunized with MOG (35–55) peptide in CFA adjuvant and mice were harvested on day 20. **D** Splenocytes were restimulated directly ex vivo and the intracellular production of IFN-γ and IL-17 by CD4^+^ T cells was determined. Pooled data are presented in (**E**). **F** Splenocytes were stimulated for 48 h with MOG (35–55) peptide and cytokine production was measured by ELISA. Data (*n* = 5 in **A**–**E**) shown are the mean ± SD. *P < 0.05, **P < 0.01, and ***P < 0.001 by an unpaired *t* test (in **A**, **C**, and **F**) and two-way ANOVA (in **B** and **E**). Data are representative of three independent experiments with similar results.

To assess whether BCL10 is the primary target of USP12 in regulating CD4^+^ T activation and function, USP12 and BCL10 double knockout (*Usp12*^−/−^ *Bcl10*^−/−^) mice were generated by crossing *Usp12*^−/−^ mice and *Bcl10*^−/−^ mice. CD4^+^ T cells from WT, *Usp12*^−/−^, *Bcl10*^−/−^, and *Usp12*^−/−^ *Bcl10*^−/−^ mice were isolated and activated in vitro using anti-CD3 and anti-CD28 antibodies. No difference in IFN-γ, TNF-α, and IL-2 expression (Fig. 7A), proliferation (Fig. 7B), and activity (Fig. 7C) was observed between BCL10 deficient groups, whether or not USP12 was deficient.

The in vivo role of BCL10 was evaluated by immunizing WT, *Usp12*^−/−^, *Bcl10*^−/−^, and *Usp12*^−/−^ *Bcl10*^−/−^ mice with MOG (35–55) peptide. Consistent with the in vitro *results*, the intracellular IFN-γ and IL-17 levels in spleens (Fig. 7E) and IFN-γ, IL-17, and TNF-α levels in serum (Fig. 7F) were significantly different between WT and *Usp12*^−/−^ mice. However, this difference disappeared when BCL10 was also deficient (Fig. 7E, F). Furthermore, the overexpression of USP12 and the enzymatically inactive USP12 (C48A) mutant protein in WT and *Bcl10*^−/−^ naïve CD4^+^ T cells showed that USP12 overexpression increased intracellular IFN-γ expression and IFN-γ, IL-17, and TNF-α secretion in WT CD4^+^ T cells, whereas the overexpression of mutant USP12 (C48A) did not (Supplementary Fig. 10A–C). Importantly, BCL10 deficiency led to decreased intracellular IFN-γ expression and IFN-γ, TNF-α, and IL-2 secretion, which was unaffected by USP12 overexpression (Supplementary Fig. 10A–C). These results confirm that BCL10 is the primary target of USP12, which regulates CD4^+^ T cell activation and function.

## Discussion

Increasing evidence suggests that DUBs play a vital role in regulating T cell responses [36–38], although the biological function of only a limited number of DUBs has been characterized, limiting DUB-based therapeutic development. In this study, we identified USP12 as a regulator of CD4^+^ T cell activation but not of CD8^+^ T cells. In CD4^+^ T cells, USP12 stabilized BCL10 through deubiquitination and activated the NF-κB signaling pathway (Supplementary Fig. 11). Although USP12 deficiency protected mice from autoimmune diseases, it resulted in lower in vivo CD4^+^ T cell responses to *L. monocytogenes* infection. Our results suggest that USP12 is an important target for regulating the adaptive immune response.

Several studies have focused on the regulatory functions of USP12 in various cell types. An earlier study suggested that USP12 deubiquitinates the androgen receptor to promote the AKT signaling pathway in prostate cancer [26]. However, in *Usp12*^−/−^ CD4^+^ T cells we did not detect changes in AKT phosphorylation compared to WT CD4^+^ T cells. These data suggest that the function of USP12 in regulating the AKT signaling pathway might depend on the cell types or activation conditions or both. A previous in vitro study showed that USP12 stabilizes the TCR complex in signaling [30]. However, we identified similar TCR-proximal signaling events, including ZAP70, LAT, and PLCγ1 phosphorylation in both WT and *Usp12*^−/−^ CD4^+^ T cells. The function of USP12 in TCR signaling pathway regulation might be different in Jurkat T cells and CD4^+^ T cells. In HeLa cells, USP12 knockdown effectively induced cell-cycle arrest and decreased BMI-1, c-Myc, and cyclin D2 transcription, suggesting that USP12 might regulate cell growth by affecting cell-cycle progression [29]. However, we found that USP12 regulated CD4^+^ T cell proliferation independent of cell-cycle progression, and dependent on IL-2 expression. A previous study showed that USP12 promoted STAT1 antiviral signaling by blocking the acetyltransferase activity of CREB-binding protein [39]. In the present study, we found that USP12 plays a vital role in the pathogenesis of autoimmune disease and bacterial infection. Thus, USP12-targeted therapies offer promise as therapeutic strategies for inflammatory diseases or for resolving pathogen infection.

Ubiquitination is a key mechanism in the CBM complex-mediated NF-κB activation pathway. The TRAF6 ubiquitin ligase mediates NF-κB activation by the K63-linked ubiquitination of BCL10 and MALT1 [40], whereas NEDD4 and Itch ubiquitin ligase promotes ubiquitination and degradation of BCL10, thus downregulating NF-κB activation [41]. In contrast, we found that in CD4^+^ T cells, USP12 promotes BCL10 stability through deubiquitination. USP12 has been shown to have very limited deubiquitylating activity in the absence of other adapter proteins such as UAF1 [35]. In CD4^+^ T cells, we also found USP12 required the presence of UAF1 to mediate stabilization of BCL10. USP46 and USP12 are >80% homologous and have been proposed to be redundant in their functions. We found these compensatory effects were also present in CD4^+^ T cells. In addition to USP12, USP9X has been implicated in BCL10 stability by removing the BCL10 ubiquitin chain to facilitate the CARMA1 association with BCL10-MALT1 [12]. The mechanism of action of USP9X is similar to that of USP12. However, we found that USP9X does not replace the USP12 function. An intriguing question is how USP12 and USP9X cooperate functionally in BCL10 regulation, and further studies are required to address this. In addition, the CBM complex plays a critical role in B cell receptor regulation, and the enforced activation of CBM signaling is adequate to drive transformed B cell expansion in vivo [42]. Thus, we speculate that USP12 might also play a role in B cell proliferation and development.

In summary, here, we identified USP12 as a positive regulator of CD4^+^ T cells, affecting both Th1 and Th17 cells in a T cell-intrinsic manner and limiting cellular activation, proliferation, and downstream IFN-γ and TNF-α expression through the BCL10-mediated NF-κB signaling pathway. Our study highlighted the critical role of USP12 in regulating the adaptive immune responses that occur during autoimmune disease and infection. Our findings suggest that USP12 might be a potential drug target for developing therapeutic interventions for use in inflammatory disease or for resolving pathogen infection.

## Supplementary information

SUPPLEMENTAL Figures
