# Supplementary material for: USP12 promotes CD4+ T cell responses through deubiquitinating and stabilizing BCL10
Source: Cell Death Differ. 2021 May 3;28(10):2857–70. doi: 10.1038/s41418-021-00787-y (PMC8481463; doi:10.1038/s41418-021-00787-y)
Supplement: Supplementary file 1 — SUPPLEMENTAL Figures [file 41418_2021_787_MOESM1_ESM.docx]

**USP12 promotes CD4^+^ T cell** **responses through deubiquitinating and stabilizing BCL10**

Yuling Fu^1#^, Peng Wang^2#^, Jingjing Zhao^3, 4#^, Yunke Tan^2^, Junli Sheng^1^, Shitong He^1^,

Xialin Du^1^, Yulan Huang^1^, Yalong Yang^1^, Jinling Li^1^, Yuxiong Cai^1^, Yuxuan Liu^1^, Shengfeng Hu^1*^

^1^Institute of Molecular Immunology, School of Laboratory Medicine and Biotechnology, Southern Medical University, Guangzhou 510515, China.

^2^Department of Emergency Medicine, Sun Yat-sen Memorial Hospital, Sun Yat-sen University, Guangzhou 510120, China.

^3^Department of Biotherapy, Sun Yat-sen University Cancer Center, Guangzhou 510060, China.

^4^State Key Laboratory of Oncology in South China, Collaborative Innovation Center for Cancer Medicine, Sun Yat-sen University Cancer Center, Guangzhou 510060, China.

# These authors contributed equally to this work.

* **Corresponding Author:**

**Shengfeng Hu**, M.D., Ph.D., Institute of Molecular Immunology, School of Laboratory Medicine and Biotechnology, Southern Medical University, Guangzhou 510515, China. E-mail: [hushengfeng@smu.edu.cn](mailto:hushengfeng@smu.edu.cn).

**Supplement figures and table**

**
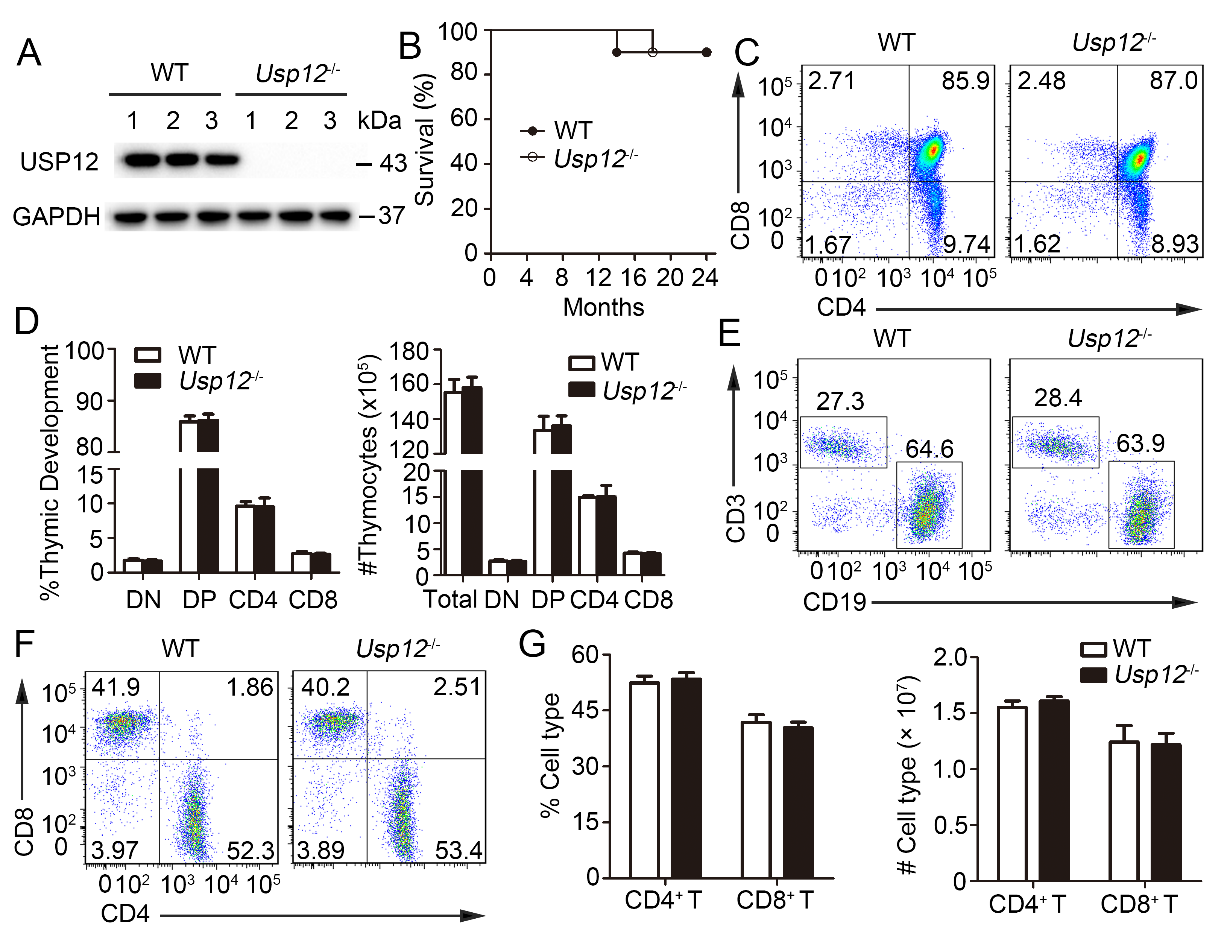
Supplement figure 1**

**Supplementary Fig. 1, Related to Fig. 1.** **USP12 did not affect thymic development. (A)** USP12 immunoblot using splenocyte from wild-type (WT) and *Usp12*^-/-^ mice. Lane represents an individual mouse. **(B)** Survival curve of WT and Usp12^-/-^ mice. **(C)** Representative expression of CD4 and CD8 from thymocytes of WT and *Usp12*^-/-^ mice (3 weeks old). **(D)** Percentages (left) and total numbers (right) of thymocytes in each stage of thymic development (n = 3 mice per genotype). DN, double negative (CD4^-^ CD8^-^); DP, double positive (CD4^+^ CD8^+^); CD4, CD4 single positive (CD4^+^ CD8^-^); CD8, CD8 single positive (CD4^-^ CD8^+^). **(E)** Representative expression of CD3^+^ T and CD19^+^ B cells from splenocytes of WT and *Usp12*^-/-^ mice (8-10 weeks old). **(F)** Representative expression of CD4^+^ T and CD8^+^ T (Gated in CD3^+^ T cells) from splenocytes of WT and *Usp12*^-/-^ mice (8-10 weeks old). **(G)** Percentages (left) and total numbers (right) of CD4^+^ T and CD8^+^ T (Gated in CD3^+^ T cells) from splenocytes of WT and *Usp12*^-/-^ mice (n = 3 mice per genotype). Data shown are the mean ±SD. Data are representative of three independent experiments with similar results.

**
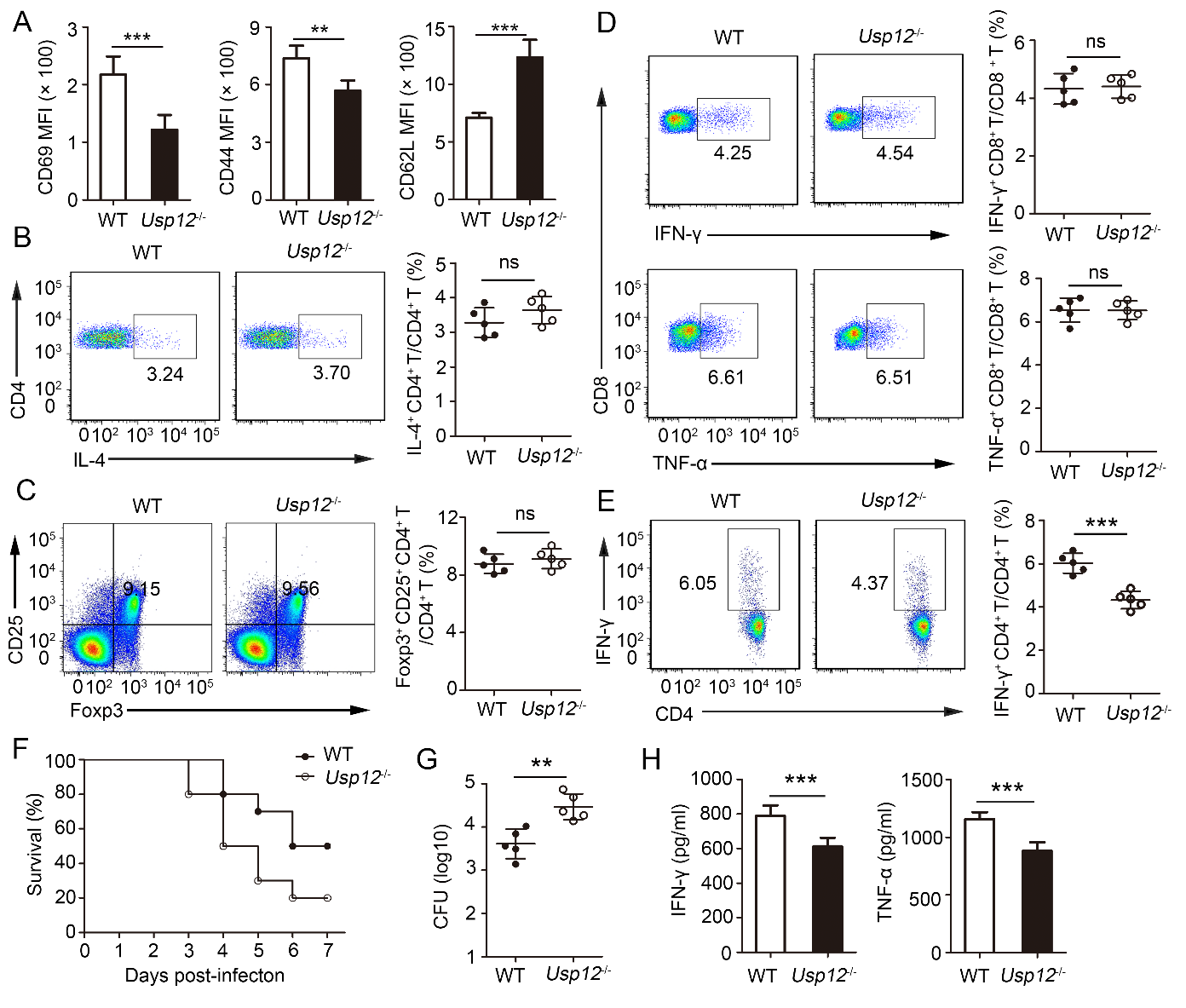
Supplement figure 2**

**Supplementary Fig. 2, Related to Fig. 1****. USP12 deficiency attenuated activation of CD4^+^ T but not CD8^+^ T cells *in vivo.* (A-D)** WT and *Usp12*^-/-^ mice were immunized with MOG(35-55) peptide in CFA adjuvant and mice were harvested on day 20. **(A)** Expression of activation markers were assessed as mean fluorescence intensity (MFI) on splenic CD4^+^ T cells (n=5). **(B)** Splenocytes were restimulated directly ex vivo and the intracellular production of IL-4 by CD4^+^ T cells was determined. Pooled data are presented in the right panel. **(C)**. Expression of CD25 and Foxp3 were detected on CD4^+^ T cells from spleens. Pooled data are presented in the right panel. **(D)** Splenocytes were restimulated directly ex vivo and the intracellular production of IFN-γ and TNF-α by CD8^+^ T cells was determined. Pooled data are presented in the right panel. **(E-H)** WT and *Usp12*^-/-^ mice were infected with *L. monocytogenes* OVA, LM-OVA (n=5 mice/group). **(E)** Splenocytes were restimulated with OVA(323-339) directly ex vivo and the intracellular production of IFN-γ by CD4^+^ T cells was determined on day 7. Pooled data are presented in the right panel. Survival curve **(F)**, liver *L. monocytogenes* titer on day 7 **(G)** and ELISA of serum IFN-γ and TNF-α concentration **(H)** of WT and *Usp12*^-/-^ mice. Data shown are the mean ±SD. ns: no significant difference, ***P* < 0.01 and ****P* < 0.001 by an unpaired *t*-test. Data are representative of three independent experiments with similar results.

**Supplement figure 3**


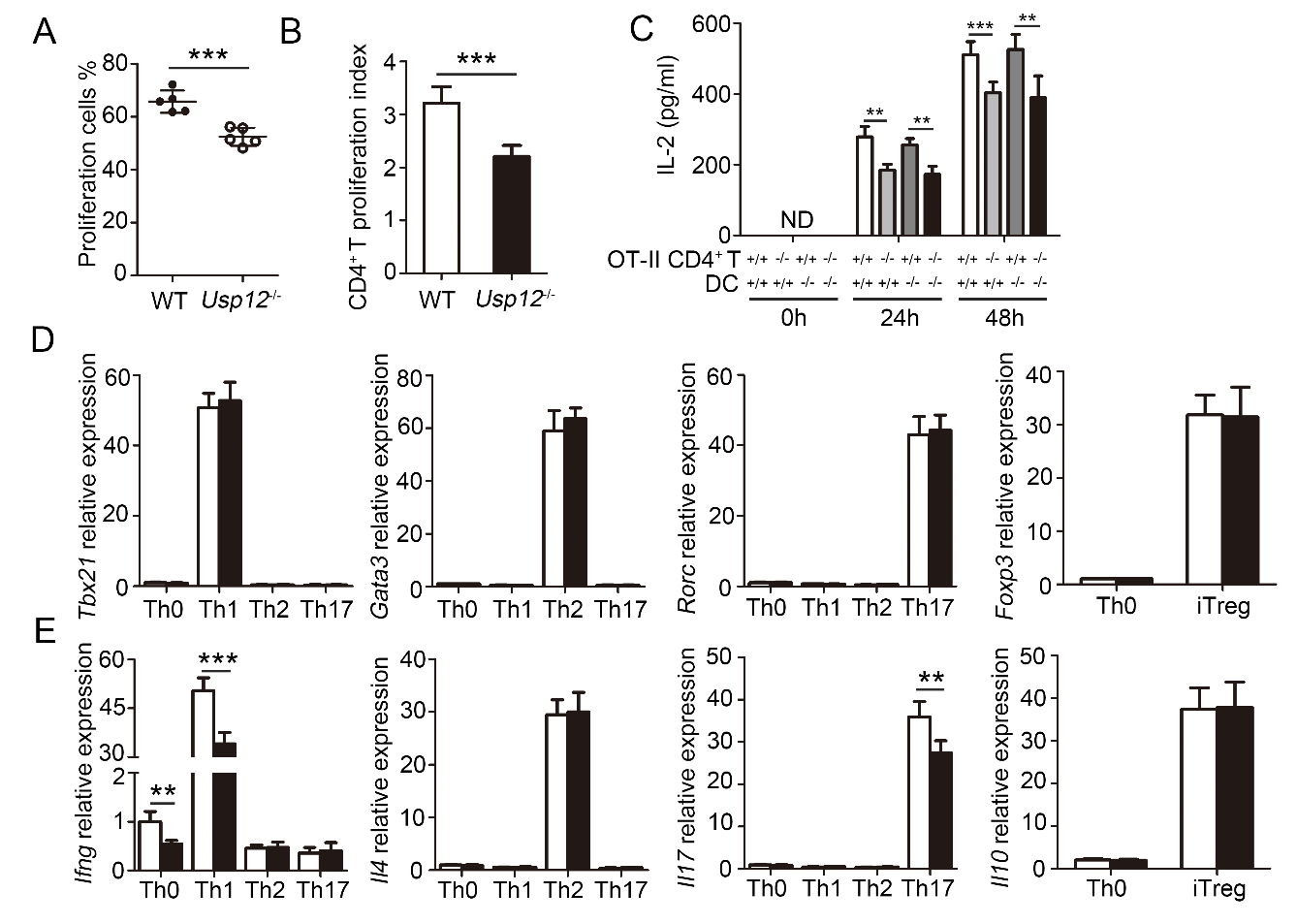
**Supplementary Fig. 3, Related to Fig. 2. CD4^+^ T cell activation *in vitro* required USP12.** **(A)** Purified naïve CD4^+^ T cells isolated from WT and *Usp12*^-/-^ mice were labeled with CFSE, stimulated with plate bound anti-CD3 (1 μg/ml) and anti-CD28 (1 μg/ml) for 72 hours and determined by flow cytometry. **(B)** The proliferation index of naïve WT and *Usp12*^-/-^ CD4^+^ T cells stimulated with plate bound anti-CD3 (1 μg/ml) and anti-CD28 (1 μg/ml) for 72 hours. **(C)** OVA(323-339) coated DCs from WT and *Usp12*^-/-^ mice were co-cultured with OT-II CD4^+^ T cells from WT OT-II or *Usp12*^-/-^ OT-II mice for indicated amounts of time. IL-2 production was measured by ELISA. +/+: WT; -/-: *Usp12*^-/-^. **(D-E)** Purified naïve CD4^+^ T cells from WT or *Usp12*^-/-^ mice were stimulated under standard Th0, Th1, Th2, Th17 or iTreg conditions and harvested on day 5. Transcription factor **(D)** and cytokine expression levels **(E)** were detected by qPCR. Data shown are the mean ±SD. ***P* < 0.01 and ****P* < 0.001 by an unpaired *t*-test (in A-B) or two-way ANOVA (in C-E). Data are representative of three independent experiments with similar results. ND: No detected.


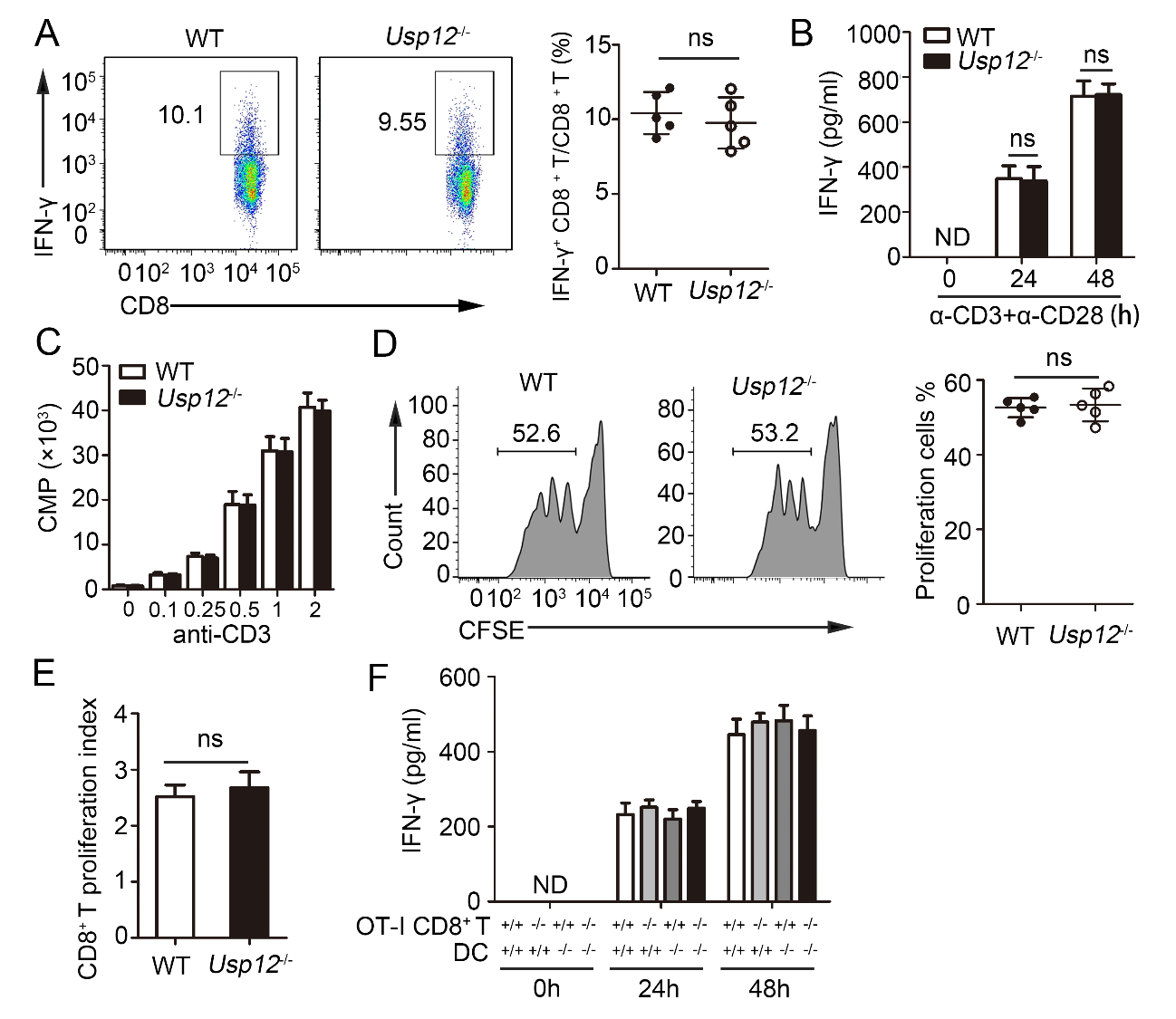
**Supplement figure 4**

**Supplementary Fig. 4, Related to Fig. 2. USP12 did not affect CD8^+^ T cell activation *in vitro*. (A)** **(A-E)** Purified naïve CD8^+^ T cells isolated from WT and *Usp12*^-/-^ mice were either not treated **(0)** or stimulated with plate bound anti-CD3 (1 μg/ml or indicated concentrations) and anti-CD28 (1 μg/ml) for 48 hours **(A and C)** or 72 hours **(D and E)** or indicated amounts of time **(B)**. **(A)** The intracellular production of IFN-γ by CD8^+^ T cells was determined. Pooled data are presented in the right panel. **(B)** IFN-γ production was measured by ELISA. **(C)** The incorporation of thymidine was measured during the final 8 hours. **(D)** Isolated purified naïve CD8^+^ T cells were labeled with CFSE, stimulated and determined by flow cytometry. Pooled data are presented in the right panel. **(E)** The proliferation index of naïve WT and *Usp12*^-/-^ CD8^+^ T cells. **(F)** OVA(257-264) coated DCs from WT and *Usp12*^-/-^ mice were co-cultured with OT-I CD8^+^ T cells from WT OT-I or *Usp12*^-/-^ OT-I mice for indicated amounts of time. IFN-γ production was measured by ELISA. +/+: WT; -/-: *Usp12*^-/-^. Data shown are the mean ±SD. ***P* < 0.01 and ****P* < 0.001 by an unpaired *t*-test (in A, D and E) or two-way ANOVA (in B, C and F). Data are representative of three independent experiments with similar results. ND: No detected.


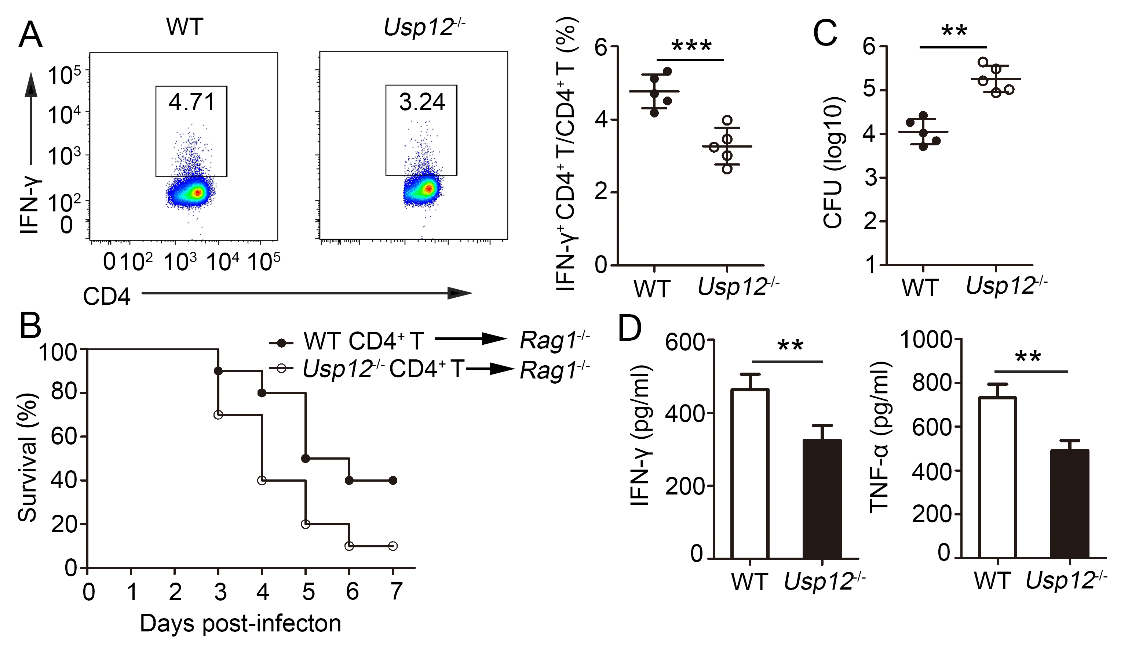
**Supplement figure 5**

**Supplementary Fig. 5, Related to Fig. 4. USP12 promoted CD4^+^ T cell responses to *L. monocytogenes* infection.** Purified WT or *Usp12*^-/-^ naïve CD4^+^ T cells were adoptively transferred into *Rag1*^-/-^ mice. 1 day later, the recipient mice were infected with *L. monocytogenes* OVA, LM-OVA. **(A)** Splenocytes were restimulated directly with OVA(323-339) ex vivo and the intracellular production of IFN-γ by CD4^+^ T cells was determined on day 7. Pooled data are presented in the right panel. Survival curve **(B)**, liver *L. monocytogenes* titer on day 7 **(C)** and ELISA of serum IFN-γ and TNF-α concentration **(H)** of recipient mice. Data shown are the mean ±SD. ***P* < 0.01 and ****P* < 0.001 by an unpaired *t*-test. Data are representative of three independent experiments with similar results.

**
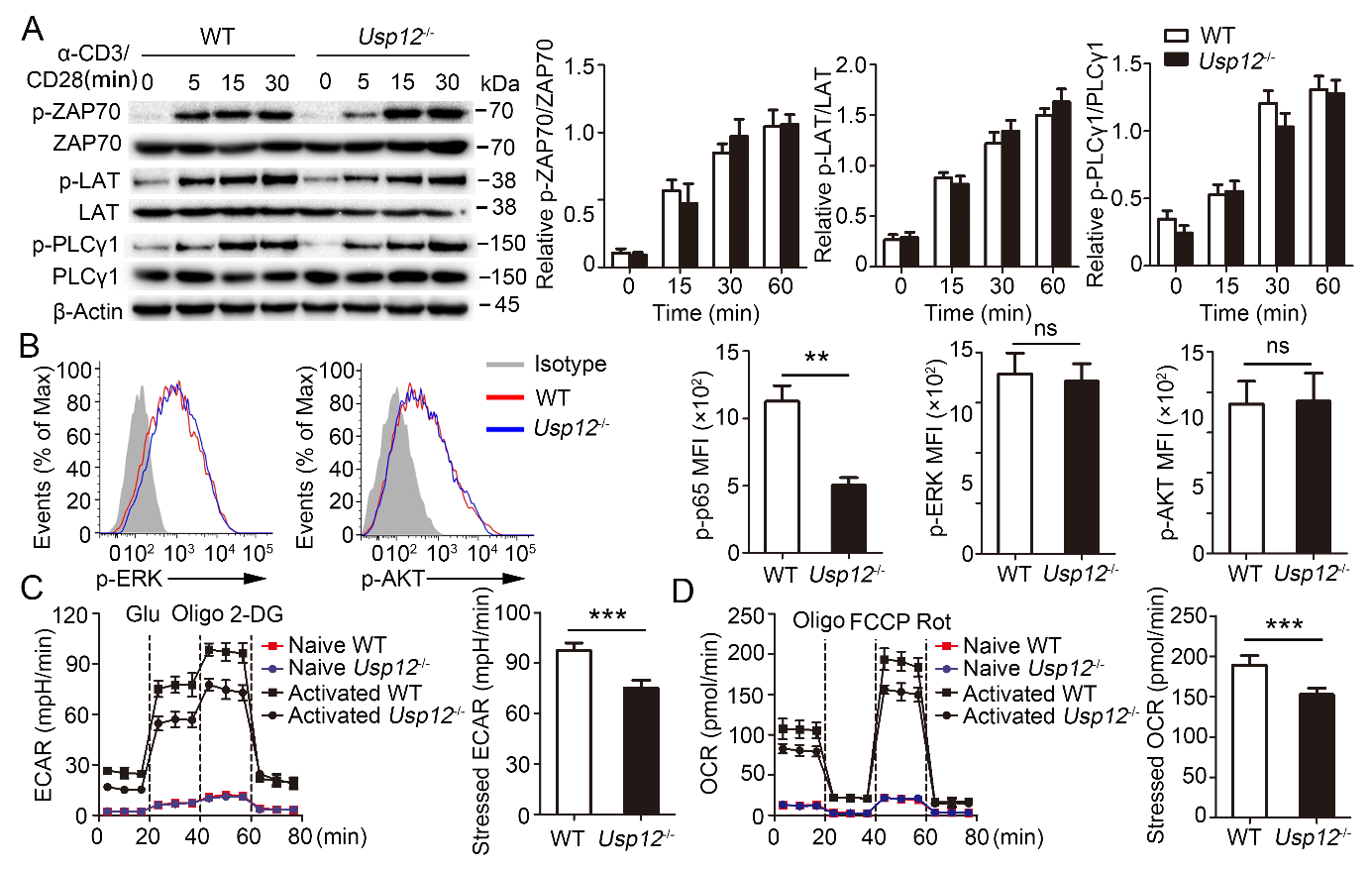
 Supplement figure 6**

**Supplementary Fig. 6, Related to Fig. 5. USP12 was dispensable for TCR-proximal signaling but indispensable for NF-κB signanling pathway. (A)** Western blot analyses of purified naïve WT and *Usp12*^-/-^ CD4^+^ T cells stimulated by anti-CD3 (1 mg/mL) and anti-CD28 (1 mg/mL) antibodies. Densitometry quantification of band intensity are presented in the right panel. **(B)** Flow cytometry analyses of phosphorylated ERK and AKT in CD4^+^ T cells sorted from spleen of WT and *Usp12*^-/-^ mice immunized with MOG(35-55) peptide in CFA adjuvant and harvested on day 20. Mean fluorescence intensity (MFI) of phosphorylated p65, ERK and AKT are presented in the right panel. **(C-D)** Purified naïve CD4^+^ T cells from WT or *Usp12*^-/-^ mice were stimulated with anti-CD3 (1 μg/ml) and anti-CD28 (1μg/ml) antibodies for 24 hours. Seahorse analysis of ECAR **(C)** and OCR **(D)** was carried out. Data shown are the mean ±SD. ***P* < 0.01 and ****P* < 0.001 by an unpaired *t*-test. Data are representative of three independent experiments with similar results.

**
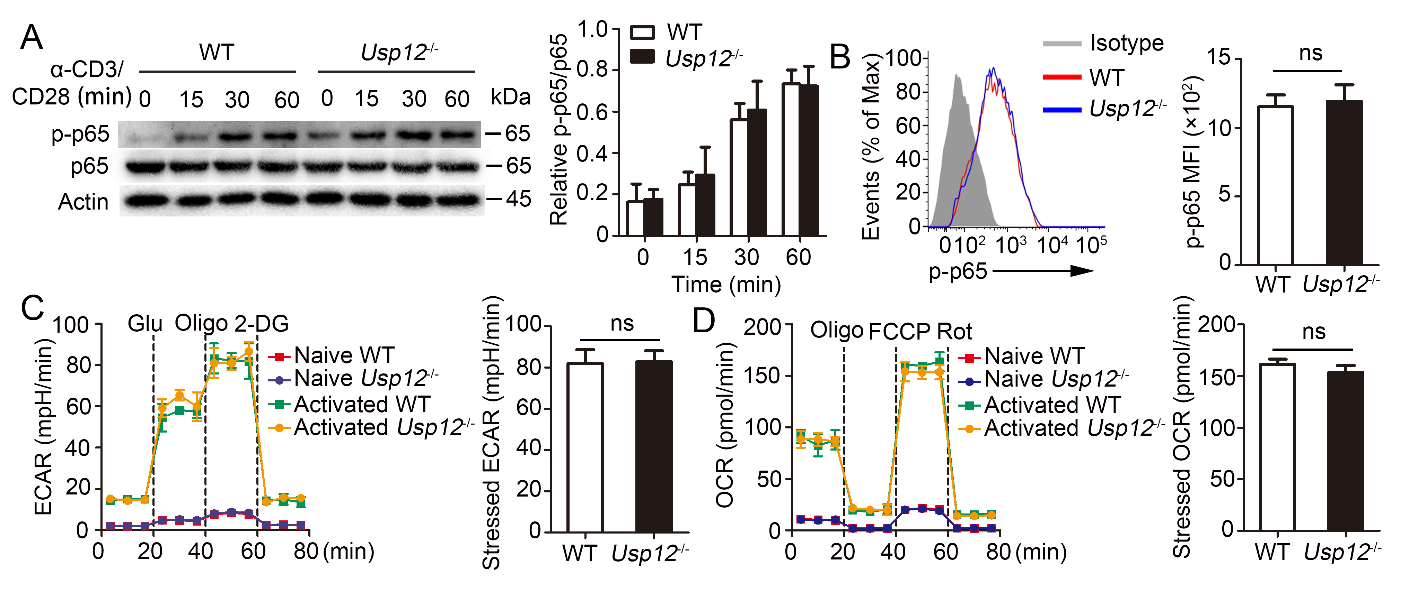
Supplement figure 7**

**Supplementary Fig. 7, Related to Fig. 5. USP12 did not affect NF-κB signaling pathway in CD8^+^ T cells. (A)** Western blot analyses of purified naïve WT and *Usp12*^-/-^ CD8^+^ T cells stimulated by anti-CD3 (1 mg/mL) and anti-CD28 (1 mg/mL) antibodies. Densitometry quantification of band intensity are presented in the right panel. **(B)** Flow cytometry analyses of phosphorylated p65 in CD8^+^ T cells sorted from spleen of WT and *Usp12*^-/-^ mice immunized with MOG(35-55) peptide in CFA adjuvant and harvested on day 20. Mean fluorescence intensity (MFI) of phosphorylated p65 was presented in the right panel. **(C-D)** Purified naïve CD8^+^ T cells from WT or *Usp12*^-/-^ mice were stimulated with anti-CD3 (1 μg/ml) and anti-CD28 (1μg/ml) antibodies for 24 hours. Seahorse analysis of ECAR **(C)** and OCR **(D)** was carried out. Data shown are the mean ±SD. Data are representative of three independent experiments with similar results.
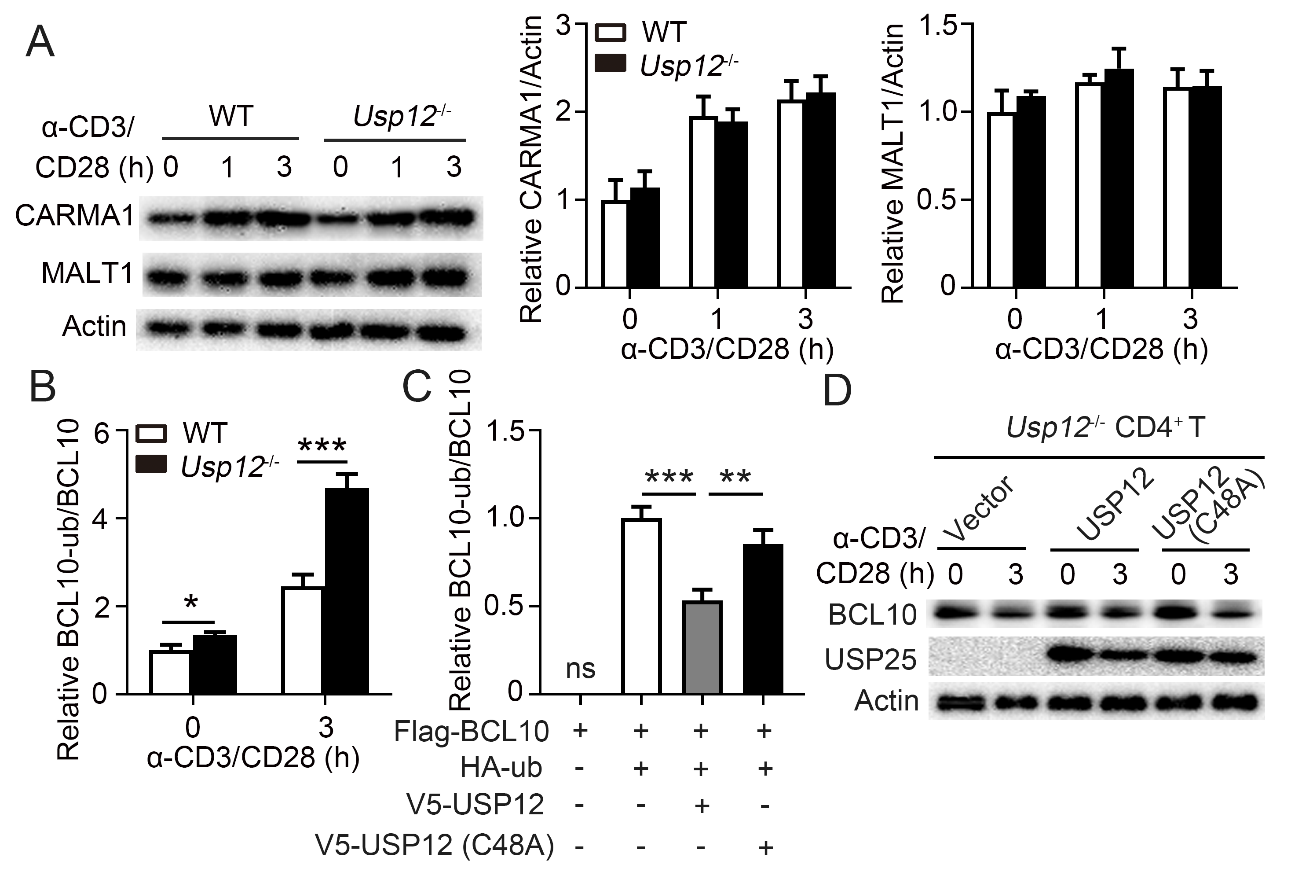
**Supplement figure 8**

**Supplementary Fig. 8, Related to Fig. 6. USP12 deubiquitinated and stabilized BCL10, but not CARMA1 and MALT1. (A)** Western blot analyses of purified naïve WT and *Usp12*^-/-^ CD4^+^ T cells stimulated by anti-CD3 (1 mg/mL) and anti-CD28 (1 mg/mL) antibodies. Densitometry quantification of band intensity are presented in the right panel. **(B)** BCL10 ubiquitinationanalysis analysis using whole-cell extracts of WT or *Usp12*^-/-^ naive CD4^+^ T cells stimulated with anti-CD3 and anti-CD28 for indicated time (**Fig6. D**). And densitometry quantification of band intensity are presented here (n=3). **(C)** BCL10 ubiquitination assays using HEK293 cells transfected with expression vectors encoding indicated proteins for 24 hours (**Fig6. G**). And densitometry quantification of band intensity are presented here (n=3). **(D)** BCL10 IB analysis using whole-cell extracts of purified naïve CD4^+^ T cells isolated from *Usp12*^-/-^ mice infected by retroviruses carrying USP12 or USP12(C48A) for 48 hours, and then stimulated with anti-CD3 (1 mg/mL) and anti-CD28 (1 mg/mL) antibodies for indicated time. Data shown are the mean ±SD. **P* < 0.05, ***P* < 0.01 and ****P* < 0.001 by an unpaired *t*-test or two-way ANOVA. Data are representative of three independent experiments with similar results.
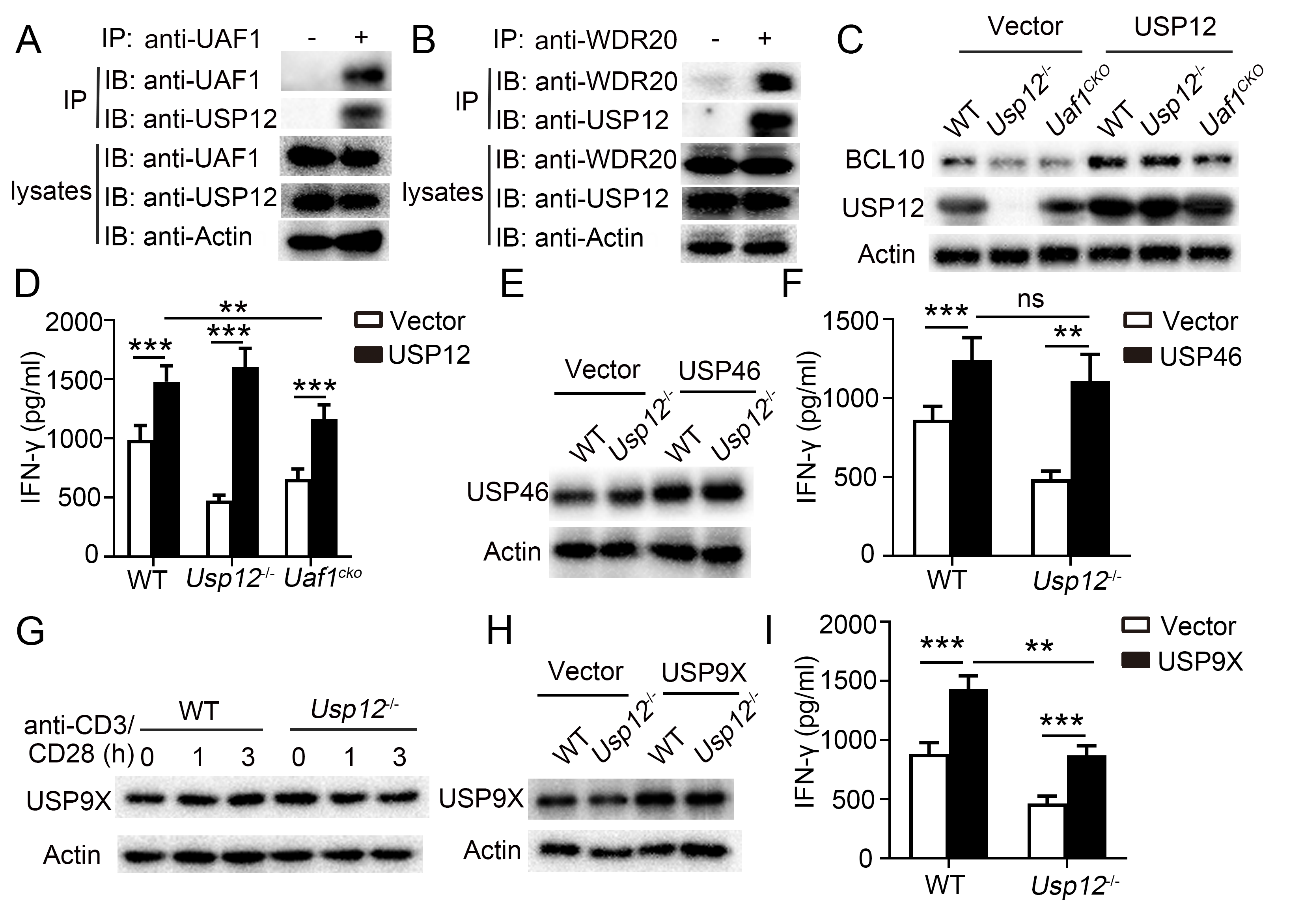
**Supplement figure 9**

**Supplementary Fig. 9, Related to Fig. 6. Usp12 mediating stabilisation of BCL10 in CD4^+^ T cells required the presence of co-factors. (A-B)** IP and IB of CD4^+^ T cells that were stimulated with anti-CD3 and anti-CD28 antibodies for 1 hours. -: control lgG; +: anti-UAF1 (WDR48) **(A)** or WDR20 **(B)**. **(C-D)** Purified naïve CD4^+^ T cells isolated from WT, *Usp12*^-/-^ or *Uaf1*^fl/fl^, CD4-Cre mice (*Uaf1^CKO^*), stimulated with anti-CD3 (1 mg/mL) and anti-CD28 (1 mg/mL) antibodies for 24 hours, and then infected by retroviruses carrying USP12 for 48 hours. (**C**) Western blot analyses of BCL10 and USP12. (**D**) IFN-γ production was measured by ELISA. **(E-F)** Purified naïve CD4^+^ T cells isolated from WT and *Usp12*^-/-^ mice, stimulated with anti-CD3 (1 mg/mL) and anti-CD28 (1 mg/mL) antibodies for 24 hours, and then infected by retroviruses carrying USP46 for 48 hours. (**E**) Western blot analyses of USP46. (**F**) IFN-γ production was measured by ELISA. (**G**) USP9X IB analysis using whole-cell extracts of WT or *Usp12*^-/-^ naive CD4^+^ T cells stimulated with anti-CD3and anti-CD28 indicated time. **(H-I)** Purified naïve CD4^+^ T cells isolated from WT and *Usp12*^-/-^ mice, stimulated with anti-CD3 (1 mg/mL) and anti-CD28 (1 mg/mL) antibodies for 24 hours, and then infected by retroviruses carrying USP46 for 48 hours. (**H**) Western blot analyses of USP46. (**I**) IFN-γ production was measured by ELISA. Data shown are the mean ±SD. ***P* < 0.01 and ****P* < 0.001 by two-way ANOVA. Data are representative of three independent experiments with similar results.

**
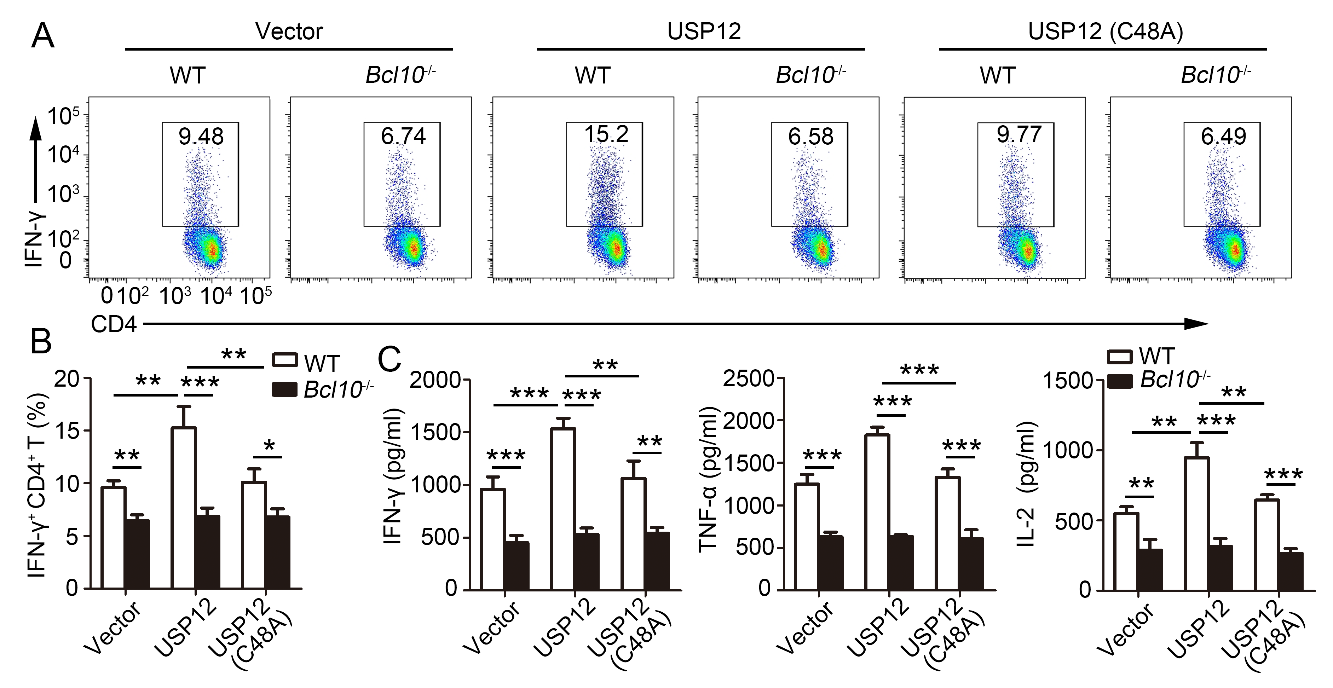
Supplement figure 10**

**Supplementary Fig. 10, Related to Fig. 7. BCL deficiency limit functions of USP12.** Purified naïve CD4^+^ T cells isolated from WT and *Bcl10*^-/-^ mice, stimulated with anti-CD3 (1 mg/mL) and anti-CD28 (1 mg/mL) antibodies for 24 hours, and then infected by retroviruses carrying USP12 or USP12(C48A) for 48 hours. **(A)** The intracellular production of IFN-γ by CD4^+^ T cells was determined. Pooled data are presented in **(B)**. **(C)** Cytokine production was measured by ELISA. Data shown are the mean ±SD. **P* < 0.05, ***P* < 0.01 and ****P* < 0.001 by two-way ANOVA. Data are representative of three independent experiments with similar results.

**Supplement figure 11**


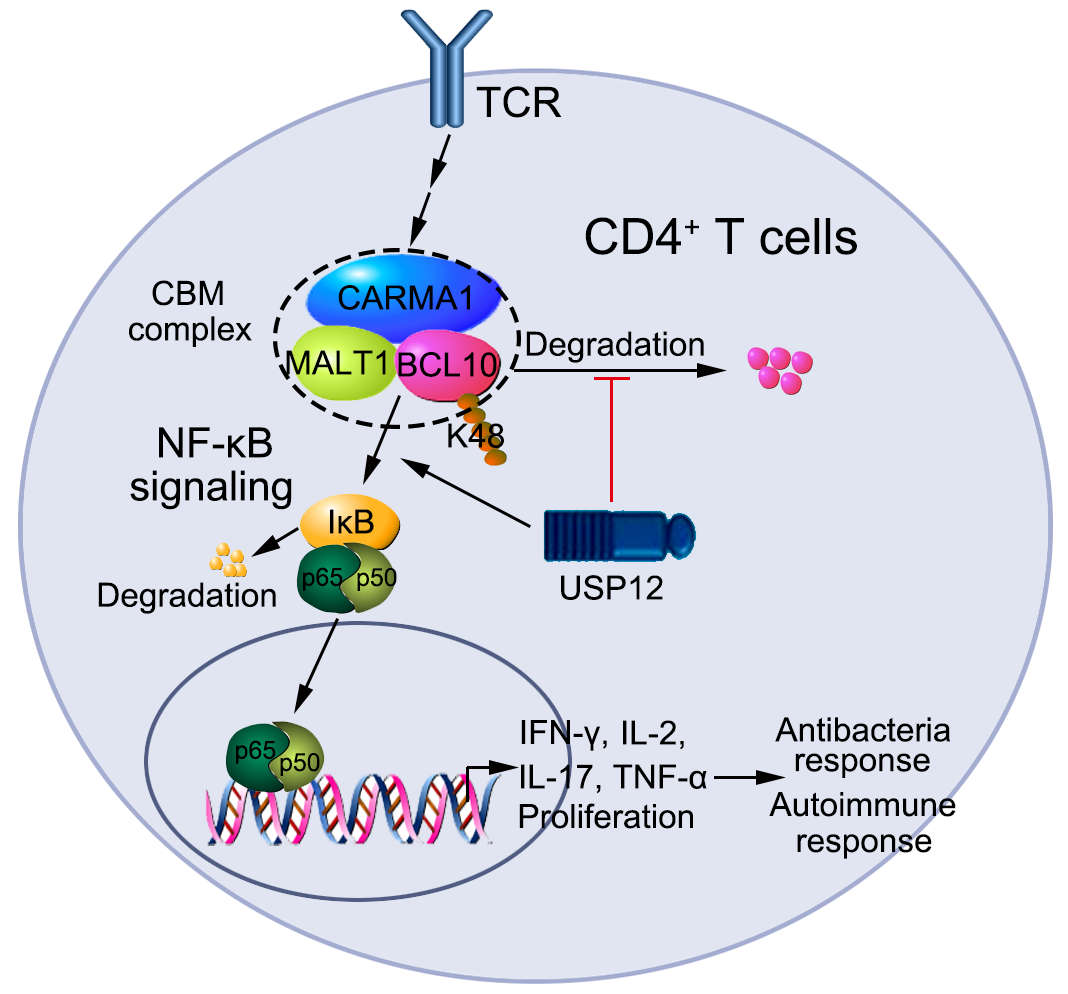


**Supplementary Fig. 11 Illustration of a model on USP12-mediated regulation of**

**CD4^+^ T responses.** Stimulated by dendritic cells or anti-CD3/28, TCR triggers CBM complex-medicated NF-κB signaling and subsequently regulates T cell survival, proliferation, and effector function. However, Constant stimulation also induces K48-linked ubiquitination and degradation of BCL10. USP12 interacts with BCL10 and removes K48-linked polyubiquitin chains from BCL10 to prevent excessive degradation of BCL10, thereby keeping CD4^+^ T response.

**Supplementary Table 1. Antibodies**

| **Antigen** | **Label** | **Clone** | **Manufacture** | **Use** |
| --- | --- | --- | --- | --- |
| CD3 | APC-eFluor® 780 | 145-2C11 | eBioscience | FCM |
| CD4 | Percp-Cy5.5 | RM4-5 | eBioscience | FCM |
| CD8 | APC | 53-6.7 | eBioscience | FCM |
| CD19 | FITC | 1D3 | eBioscience | FCM |
| IFN-γ | eFluor 450 | XMG1.2 | eBioscience | FCM |
| IL-17A | FITC | eBio17B7 | eBioscience | FCM |
| TNF-α | PE | MP6-XT22 | eBioscience | FCM |
| IL-2 | APC | JES6-5H4 | eBioscience | FCM |
| CD69 | PE | H1.2F3 | eBioscience | FCM |
| CD44 | FITC | IM7 | eBioscience | FCM |
| CD62L | PE | MEL-14 | eBioscience | FCM |
| IL-4 | PE-Cy7 | 11B11 | eBioscience | FCM |
| Foxp3 | FITC | FJK-16s | eBioscience | FCM |
| CD25 | PE | PC61.5 | eBioscience | FCM |
| USP12 |  | polyclonal | Sigma | WB |
| p-ZAP70 |  | 65E4 | CST | WB |
| ZAP70 |  | D1C10E | CST | WB |
| p-LAT |  | E3U6 | CST | WB |
| LAT |  | E3S5L | CST | WB |
| p-PLCγ1 |  | D9H10 | CST | WB |
| PLCγ1 |  | D6M9S | CST | WB |
| p-p65 |  | 93H1 | CST | WB |
| p65 |  | D14E12 | CST | WB |
| p-ERK |  | D13.14.4E | CST | WB |
| ERK |  | 137F5 | CST | WB |
| p-AKT |  | D9E | CST | WB |
| AKT |  | C67E7 | CST | WB |
| Ubiquitin |  | P4D1 | CST | WB |
| FLAG |  | D6W5B | CST | WB |
| HA |  | C29F4 | CST | WB |
| V5 |  | D3H8Q | CST | WB |
| Myc |  | 9B11 | CST | WB |
| UAF1(WDR48) |  | E-4 | Santa Cruz | WB |
| WDR20 |  | 38K | Santa Cruz | WB |
| USP9X |  | D4Y7W | CST | WB |
| USP46 |  | PA5-110560 | Thermo Fisher | WB |
| β-Actin |  | D6A8 | CST | WB |

| **Gene** | **Forward primer** | **Reverse primer** |
| --- | --- | --- |
| *mActb* | CATTGCTGACAGGATGCAGAAGG | TGCTGGAAGGTGGACAGTGAGG |
| *mTbx21* | CCACCTGTTGTGGTCCAAGTTC | CCACAAACATCCTGTAATGGCTTG |
| *mGata3* | CCTCTGGAGGAGGAACGCTAAT | GTTTCGGGTCTGGATGCCTTCT |
| *mRorc* | GTGGAGTTTGCCAAGCGGCTTT | CCTGCACATTCTGACTAGGACG |
| *mFoxp3* | CCTGGTTGTGAGAAGGTCTTCG | TGCTCCAGAGACTGCACCACTT |
| *mIfng* | CAGCAACAGCAAGGCGAAAAAGG | TTTCCGCTTCCTGAGGCTGGAT |
| *mIl4* | ATCATCGGCATTTTGAACGAGGTC | ACCTTGGAAGCCCTACAGACGA |
| *mIl17a* | CAGACTACCTCAACCGTTCCAC | TCCAGCTTTCCCTCCGCATTGA |
| *mIl10* | CGGGAAGACAATAACTGCACCC | CGGTTAGCAGTATGTTGTCCAGC |

**Supplementary Table 2. Gene-specific primers used for qRT-PCR**
